# Radiotherapy-induced oxidative stress and mitochondrial dysfunction: potential contributions of toxic elements and associated biomarkers—a systematic review

**DOI:** 10.3389/fpubh.2026.1969155

**Published:** 2026-10-14

**Authors:** Amjad Ahmed Saleh Alharkan, Ashraf Mahmoud Emara, Samia Soliman Barghash

**Affiliations:** 1 Department of Pharmacology and Toxicology, Qassim University, Buraidah, Saudi Arabia; 2 Department of Forensic Medicine and Clinical Toxicology, Faculty of Medicine for Girls, Al-Azhar University, Cairo, Egypt

**Keywords:** radiotherapy, radiation-induced toxicity, oxidative stress, mitochondrial dysfunction, toxic elements, heavy metals, biomarkers, inductively coupled plasma mass spectrometry (ICP-MS)

## Abstract

**Introduction:**

Radiotherapy is a cornerstone of cancer treatment; however, radiation-induced injury to normal tissues remains an important limitation. Ionizing radiation generates reactive oxygen species (ROS) through direct and indirect mechanisms, including water radiolysis, thereby contributing to oxidative stress, mitochondrial dysfunction, inflammation, and normal-tissue injury. This systematic review synthesizes clinical and experimental evidence concerning the molecular mechanisms of radiotherapy-induced oxidative and mitochondrial injury, with particular attention to the potential modifying role of toxic-element exposure and associated biomarkers.

**Methods:**

The available clinical and experimental evidence on radiotherapy-induced oxidative stress, mitochondrial dysfunction, and the potential contribution of toxic-element exposure was systematically reviewed. Particular consideration was given to lead (Pb), cadmium (Cd), arsenic (As), mercury (Hg), chromium (Cr), nickel (Ni), and aluminum (Al), as well as biomarkers of oxidative, antioxidant, mitochondrial, and cellular stress. The potential utility of inductively coupled plasma mass spectrometry (ICP-MS) for quantifying toxic-element burden was also evaluated.

**Results:**

Experimental and limited clinical evidence suggests that exposure to Pb, Cd, As, Hg, Cr, Ni, and Al may alter antioxidant defenses, redox homeostasis, and mitochondrial function and could therefore influence cellular responses to radiotherapy. However, direct clinical evidence demonstrating that toxic-element burden independently or synergistically increases radiotherapy toxicity remains limited. Malondialdehyde (MDA), catalase (CAT), and glutathione peroxidase (GPx) provide complementary information regarding lipid peroxidation and antioxidant defense, whereas growth differentiation factor-15 (GDF-15) represents a broader stress-responsive marker that may reflect mitochondrial and cellular stress but is not radiation-specific. ICP-MS provides sensitive multi-element quantification and may facilitate investigation of toxic-element burden in patients receiving radiotherapy.

**Discussion and Conclusion:**

The evidence supports an integrated mechanistic framework linking radiation-induced oxidative stress, mitochondrial dysfunction, inflammatory signaling, and potential toxic-element-related susceptibility. Nevertheless, the proposed toxic-element “double-hit” model remains a testable hypothesis rather than a clinically validated phenomenon. Prospective longitudinal studies integrating toxic-element measurements, oxidative and mitochondrial biomarkers, radiation dosimetry, and clinically defined toxicity outcomes are required to establish its clinical relevance.

## 1 Introduction

Cancer remains a leading global cause of morbidity and mortality, necessitating continued efforts to improve therapeutic efficacy while minimizing treatment-related toxicity (1, 2). Advances in cancer therapy have substantially improved disease control and survival; however, treatment-associated adverse effects remain significant determinants of treatment tolerance, quality of life, and long-term outcomes (3, 4). Consequently, contemporary oncology seeks to characterize the molecular mechanisms underlying treatment-related tissue injury and the biological factors that may explain interindividual variation in treatment response.

Radiotherapy (RT) is a cornerstone of modern cancer treatment and is used with curative, adjuvant, neoadjuvant, and palliative intent across a broad range of malignancies (5, 6). Advances in radiation planning and delivery, including intensity-modulated radiotherapy, image-guided radiotherapy, stereotactic techniques, volumetric-modulated arc therapy, and proton therapy, have improved dose conformity and tumor targeting while reducing unnecessary irradiation of surrounding tissues (7, 8). Nevertheless, radiation-induced normal-tissue toxicity remains an important clinical limitation and may affect treatment tolerance, quality of life, and long-term outcomes. Understanding the molecular pathways responsible for radiation-associated injury is therefore essential for improving toxicity prediction, prevention, and individualized cancer care.

The biological effects of ionizing radiation occur through both direct and indirect mechanisms. Direct effects result from energy deposition in DNA and other critical cellular macromolecules, whereas indirect effects arise primarily from radiolysis of intracellular water and subsequent generation of reactive oxygen species (ROS), including hydroxyl radicals (•OH), superoxide (O₂•^−^), and hydrogen peroxide (H₂O₂) (9–11). These reactive species can promote lipid peroxidation, protein oxidation, DNA damage, and disruption of redox-sensitive signaling pathways (9–11). Importantly, radiation-induced ROS are not exclusively detrimental: oxidative stress contributes to tumor-cell killing and therapeutic efficacy, whereas excessive or persistent redox imbalance can contribute to normal-tissue injury (12, 13). The biological consequences of radiation depend on the magnitude and duration of oxidative stress, cellular antioxidant capacity, tissue context, and the ability of cells to restore redox homeostasis (Figure 1).

**Figure 1:**
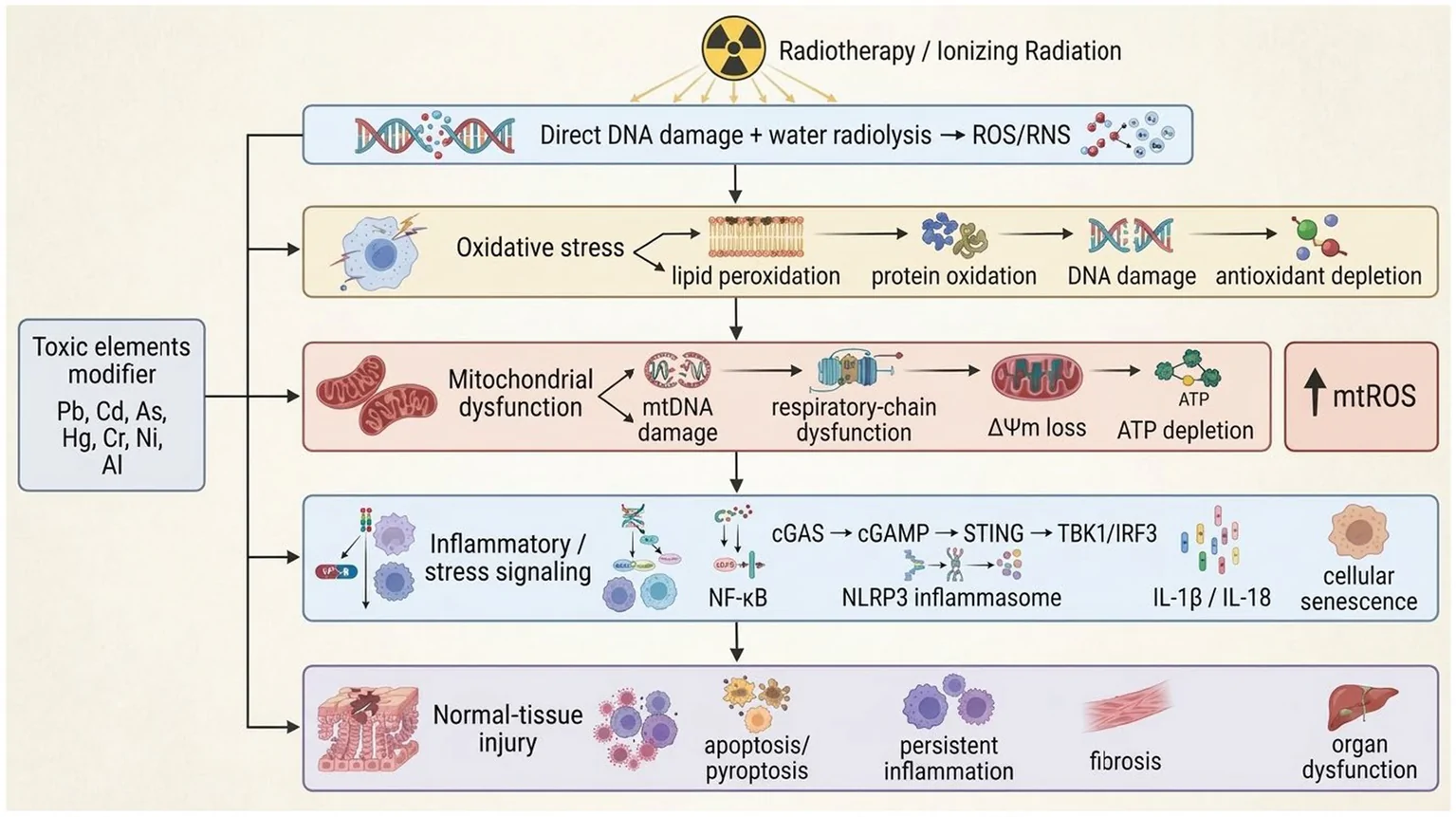
Mechanistic framework of radiotherapy-induced oxidative and nitrosative stress and mitochondrial dysfunction, with the potential modifying contribution of toxic-element exposure. Ionizing radiation induces direct DNA damage and water radiolysis, generating reactive oxygen and nitrogen species (ROS/RNS). Excess ROS/RNS drives oxidative stress, leading to lipid peroxidation, protein oxidation, DNA damage, and antioxidant depletion. This cascades into mitochondrial dysfunction, characterized by mtDNA damage, respiratory-chain dysfunction, loss of mitochondrial membrane potential (ΔΨ_m_), ATP depletion, and elevated mitochondrial ROS (increase mtROS). Subsequent inflammatory and stress signaling involves NFκB activation, the cGAS cGAMP STING TBK1/IRF3 axis, NLRP3 inflammasome activation, release of IL-1β/IL-18, and cellular senescence. Ultimately, these pathways culminate in normal-tissue injury, encompassing apoptosis/pyroptosis, persistent inflammation, fibrosis, and organ dysfunction. Chronic exposure to toxic elements (Pb, Cd, As, Hg, Cr, Ni, Al) serves as a modifying input that converges on these pathways to exacerbate oxidative stress, deplete antioxidants, and worsen mitochondrial dysfunction. The proposed interaction between toxic-element burden and radiotherapy remains a mechanistic hypothesis requiring prospective clinical validation. Created by the authors using FigureLabs (https://chat.figurelabs.ai/).

Mitochondria are particularly important in radiation-induced cellular injury because they are both targets of oxidative damage and major sources of intracellular ROS. Radiation-associated injury to mitochondrial respiratory-chain components and mitochondrial DNA (mtDNA) may increase electron leakage and mitochondrial ROS production, potentially establishing a self-reinforcing cycle of oxidative stress and mitochondrial impairment (14–19). Persistent mitochondrial dysfunction may impair oxidative phosphorylation, reduce ATP production, alter mitochondrial membrane potential, disrupt cellular bioenergetics, and activate intrinsic apoptotic pathways (15–22). Mitochondrial injury may additionally interact with inflammatory and tissue-remodeling pathways, contributing to chronic inflammation, cellular senescence, fibrosis, genomic instability, and delayed tissue injury (18–22). Thus, mitochondrial dysfunction represents an important mechanistic link between early radiation-associated oxidative stress and subsequent cellular and tissue-level consequences.

The relationship between oxidative stress and radiotherapy response is further influenced by the metabolic characteristics of cancer cells. Malignant cells frequently exhibit altered metabolism, increased basal ROS production, mitochondrial abnormalities, and enhanced dependence on adaptive antioxidant systems (15, 22). Radiotherapy may exploit this altered redox state by increasing ROS beyond the threshold compatible with tumor-cell survival, thereby promoting apoptosis and other forms of radiation-induced cell death (13, 15, 22). Conversely, surviving tumor cells may activate antioxidant and stress-response pathways, including nuclear factor erythroid 2-related factor 2 (Nrf2)-dependent signaling and glutathione- and thioredoxin-mediated defenses, potentially contributing to radioresistance (13, 23). These observations highlight the dual role of oxidative stress in radiotherapy: it contributes to tumor-cell killing while also participating in normal-tissue toxicity and adaptive cellular responses.

Environmental and occupational exposure to toxic elements represents a potentially important but comparatively underexplored modifier of this biological response. Lead (Pb), cadmium (Cd), arsenic (As), mercury (Hg), chromium (Cr), nickel (Ni), and aluminum (Al) have been associated with oxidative stress, impaired antioxidant defenses, altered mitochondrial function, and cellular injury (24–37). However, these elements should not be considered biologically equivalent. Their effects depend on chemical form, dose, route and duration of exposure, tissue distribution, biological half-life, and individual susceptibility. Several toxic elements can disrupt glutathione homeostasis, interact with sulfhydryl-containing proteins, interfere with mitochondrial respiratory function, and promote ROS generation and lipid peroxidation (24–37). These mechanisms provide a biologically plausible basis for investigating whether pre-existing toxic-element exposure may modify cellular responses to subsequent radiation exposure (Table 1).

**Table 1:** Toxic-element-specific mechanisms contributing to oxidative stress and mitochondrial dysfunction.

Toxic element	Principal oxidative/redox mechanisms	Mitochondrial and cellular mechanisms	Major reported biological consequences	References
Lead (Pb)	Inhibition of sulfhydryl-containing antioxidant enzymes; disruption of antioxidant defense; increased ROS generation; enhanced lipid peroxidation	Mitochondrial dysfunction; interference with calcium-dependent intracellular signaling; impairment of cellular homeostasis	Oxidative injury, mitochondrial stress, cellular dysfunction, and tissue toxicity	(30, 37)
Cadmium (Cd)	Glutathione (GSH) depletion; inhibition of antioxidant defenses; increased ROS generation; enhanced lipid and protein oxidation	Mitochondrial respiratory-chain inhibition; mitochondrial dysfunction; activation of inflammatory signaling pathways	Oxidative injury, mitochondrial impairment, inflammation, and cellular toxicity	(31, 37)
Arsenic (As)	Redox imbalance; increased ROS/RNS generation; lipid peroxidation; impairment of antioxidant defenses	Disruption of mitochondrial respiration and cellular energy metabolism; mitochondrial dysfunction; DNA damage	Oxidative stress, genomic injury, mitochondrial dysfunction, inflammation, and cellular toxicity	(33, 37)
Mercury (Hg)	High affinity for thiol-containing groups; inhibition of antioxidant enzymes; increased ROS/RNS generation; depletion of cellular antioxidant capacity	Mitochondrial membrane disruption; impaired mitochondrial function; mitochondrial oxidative injury	Oxidative stress, mitochondrial injury, enzyme dysfunction, and cellular toxicity	(32, 37)
Chromium (Cr)	Redox cycling and ROS generation; lipid peroxidation; oxidative DNA damage; impaired antioxidant defenses	Mitochondrial dysfunction and cellular energy disturbance; interaction with DNA and proteins	DNA damage, genomic instability, oxidative injury, carcinogenicity, and cellular toxicity	(34, 37)
Nickel (Ni)	Inhibition of antioxidant defenses; increased ROS generation; lipid peroxidation; protein oxidation	Mitochondrial impairment and altered cellular energy metabolism; oxidative injury	Oxidative stress, DNA damage, genomic instability, inflammation, and cellular toxicity	(35, 37)
Aluminum (Al)	Increased oxidative stress; enhanced lipid peroxidation; reduced GSH and antioxidant defense; protein oxidation	Mitochondrial dysfunction; altered cellular energy metabolism; disruption of cellular homeostasis	Oxidative injury, altered cellular metabolism, mitochondrial stress, and tissue toxicity	(36, 37)

ROS, reactive oxygen species; RNS, reactive nitrogen species; GSH, glutathione; DNA, deoxyribonucleic acid.

The mechanisms listed represent the principal pathways reported in the literature cited in this review. The toxic elements should not be considered mechanistically equivalent, and the magnitude and clinical relevance of their effects depend on chemical species, exposure dose, duration, route of exposure, tissue distribution, and individual susceptibility. Direct evidence demonstrating that these toxic elements independently or synergistically increase radiotherapy toxicity in cancer patients remains limited.

The potential interaction between toxic-element burden and radiotherapy can be conceptualized as a ‘double-hit’ framework. In this model, chronic exposure to toxic elements may establish a pre-existing state of oxidative and mitochondrial stress, potentially reducing cellular antioxidant and bioenergetic reserve before radiotherapy. Subsequent radiation exposure could impose an additional oxidative challenge and thereby increase the overall burden of ROS-mediated cellular injury. However, this interaction should currently be regarded as a mechanistic hypothesis rather than a clinically validated phenomenon. Although experimental evidence supports substantial overlap between the pathways activated by toxic elements and ionizing radiation, direct prospective clinical evidence demonstrating additive or synergistic effects of toxic-element burden and radiotherapy remains limited. Accordingly, the proposed double-hit framework should be interpreted cautiously, with a clear distinction between established biological mechanisms and emerging hypotheses (see Figure 2).

**Figure 2:**
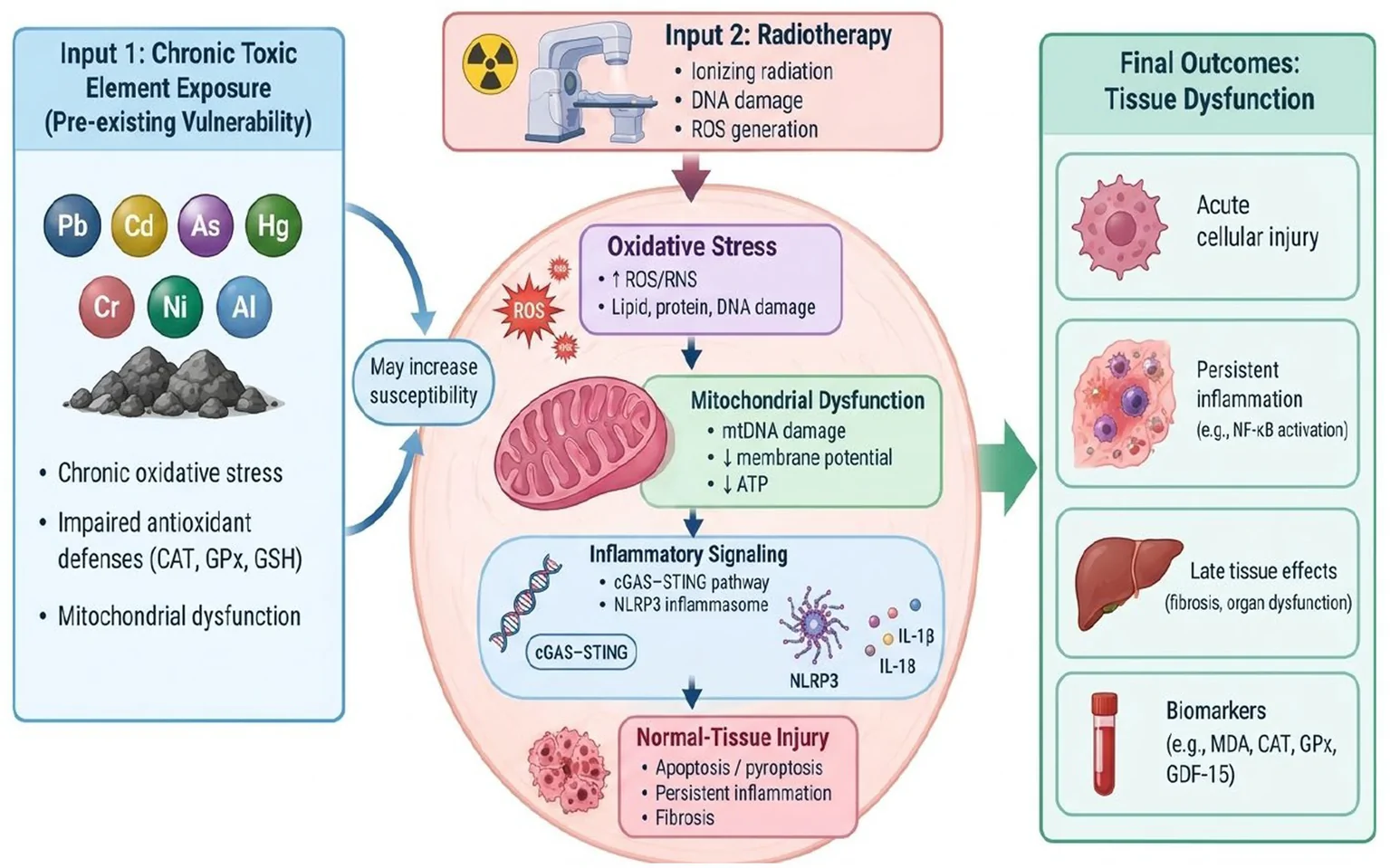
Illustrates the proposed “double-hit” framework, in which chronic toxic-element exposure establishes a pre-existing state of oxidative and mitochondrial stress that may lower the cellular threshold for radiation-induced injury. Created by the authors using FigureLabs (https://chat.figurelabs.ai/).

The potential interaction between toxic-element burden and radiotherapy can be conceptualized as a ‘double-hit’ framework. In this model, chronic exposure to toxic elements may establish a pre-existing state of oxidative and mitochondrial stress, potentially reducing cellular antioxidant and bioenergetic reserve before radiotherapy. Subsequent radiation exposure could impose an additional oxidative challenge and thereby increase the overall burden of ROS-mediated cellular injury. However, this interaction must be regarded strictly as a mechanistic hypothesis rather than a clinically validated phenomenon. Although experimental evidence supports substantial overlap between the pathways activated by toxic elements and ionizing radiation, direct prospective clinical evidence demonstrating additive or synergistic effects of toxic-element burden and radiotherapy remains limited. Accordingly, the proposed double-hit framework should be interpreted cautiously, with a clear distinction between established biological mechanisms and emerging hypotheses.

Biomarkers of oxidative and cellular stress may provide complementary tools for investigating these relationships. Malondialdehyde (MDA), a product of lipid peroxidation, is widely used as an indicator of oxidative membrane damage, whereas catalase (CAT) and glutathione peroxidase (GPx) represent important components of enzymatic antioxidant defense (38–41). Changes in these markers may provide information on oxidative damage and endogenous antioxidant capacity, but their interpretation can be influenced by cancer type, radiotherapy regimen, concomitant chemotherapy, nutritional status, smoking, systemic disease, and analytical methodology (38–41). Importantly, MDA, CAT, and GPx should not be interpreted as specific indicators of mitochondrial dysfunction; rather, they reflect distinct components of the broader redox response.

Growth differentiation factor-15 (GDF-15) represents a distinct category of stress-responsive biomarker. GDF-15 is a cytokine belonging to the transforming growth factor-β superfamily and may be induced in response to mitochondrial stress, oxidative injury, inflammation, cellular senescence, and metabolic disturbance (42–45). Elevated GDF-15 concentrations have been reported in mitochondrial disorders and several chronic diseases, supporting its potential relevance as a marker of cellular stress (42–45). Nevertheless, GDF-15 is not specific to mitochondrial dysfunction, and its elevation may reflect several overlapping pathological processes. Its potential application in monitoring radiation-associated mitochondrial or cellular stress should therefore be regarded as an emerging research area requiring prospective clinical validation (Figure 3).

**Figure 3:**
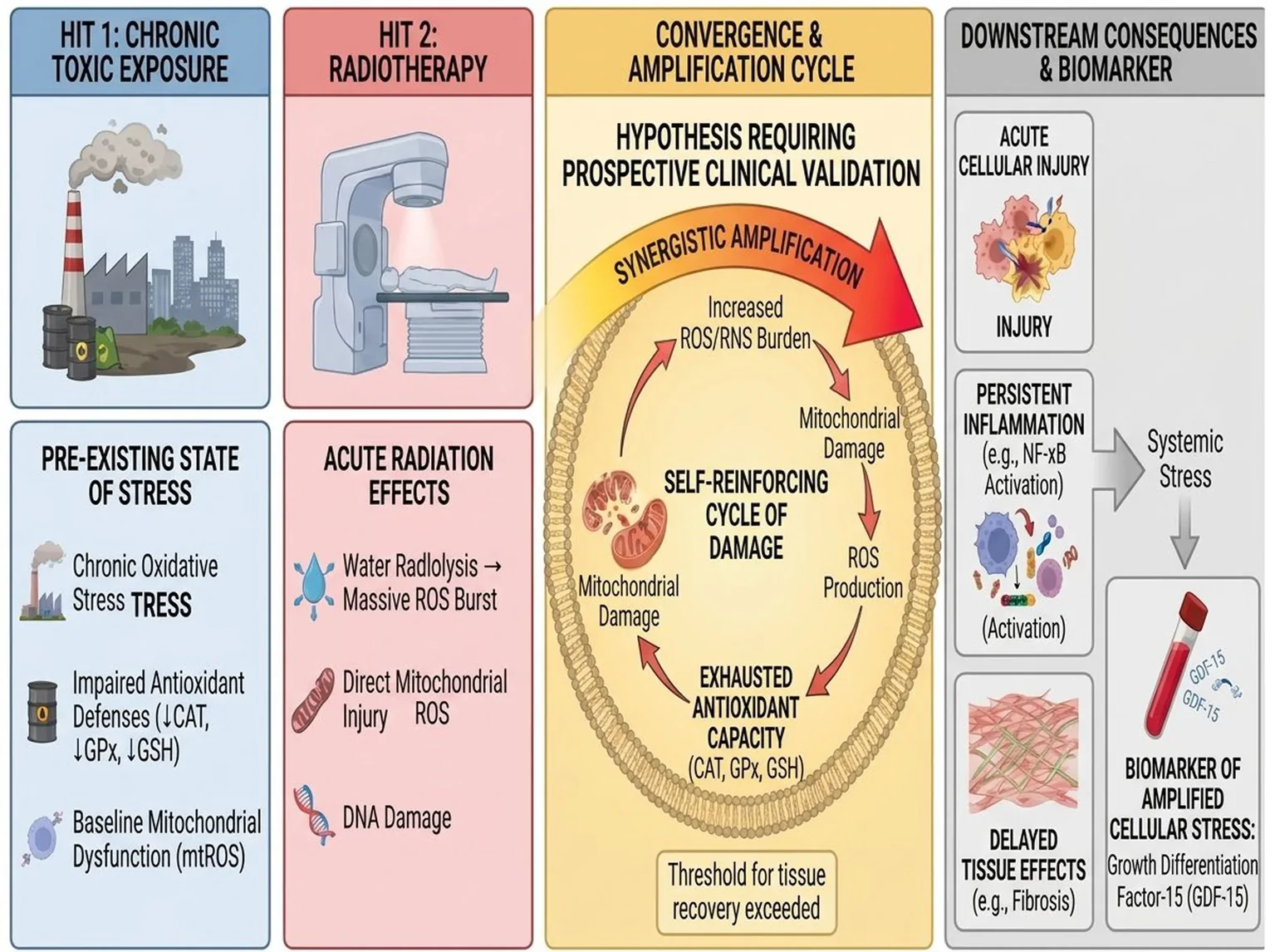
Proposed framework for potential interaction between chronic toxic-element exposure and radiotherapy-associated tissue injury. Created by the authors using FigureLabs (https://chat.figurelabs.ai/). Hit 1 (left): Chronic environmental or occupational exposure to toxic elements (lead, cadmium, arsenic, mercury, chromium, nickel, and aluminum) establishes a pre-existing state of cellular stress characterized by chronic oxidative stress, impaired enzymatic antioxidant defenses (reduced catalase [CAT], glutathione peroxidase [GPx], and glutathione [GSH]), and baseline mitochondrial dysfunction with elevated mitochondrial reactive oxygen species (mtROS). Hit 2 (center-left): Radiotherapy generates a massive burst of reactive oxygen species (ROS) through water radiolysis, produces direct mitochondrial injury, and causes DNA damage. Convergence and amplification (center): Potential convergence of radiation-induced oxidative stress with pre-existing toxic-element-associated cellular stress may increase the overall oxidative and mitochondrial burden; whether this interaction produces clinically meaningful additive or synergistic toxicity remains unestablished. This initiates a self-reinforcing cycle in which increased ROS/reactive nitrogen species (RNS) burden causes mitochondrial damage, which in turn amplifies ROS production; concurrently, antioxidant capacity becomes exhausted, further propagating oxidative injury. Downstream consequences (right): The amplified stress cascade results in acute cellular injury, persistent inflammation (e.g., nuclear factor kappa-B [NF-κB] activation), and delayed tissue effects such as fibrosis. Growth differentiation factor-15 (GDF-15) is proposed as a systemic biomarker reflecting this amplified cellular and mitochondrial stress. This model is presented as a biologically plausible mechanistic framework; direct prospective clinical evidence demonstrating additive or synergistic toxicity from combined toxic-element burden and radiotherapy in cancer patients remains limited and requires further validation.

Accurate assessment of toxic-element burden is another critical component of this research framework. Inductively coupled plasma mass spectrometry (ICP-MS) is a highly sensitive analytical platform widely used for quantitative determination of trace and toxic elements in biological specimens (46, 47). Its capacity for multi-element detection and its low detection limits make it particularly suitable for investigating environmental and occupational exposure. When appropriately combined with validated assays for oxidative and cellular stress biomarkers, ICP-MS may facilitate integrated assessment of relationships among toxic-element burden, redox imbalance, mitochondrial stress, and radiation-associated tissue injury (see Figure 4).

**Figure 4:**
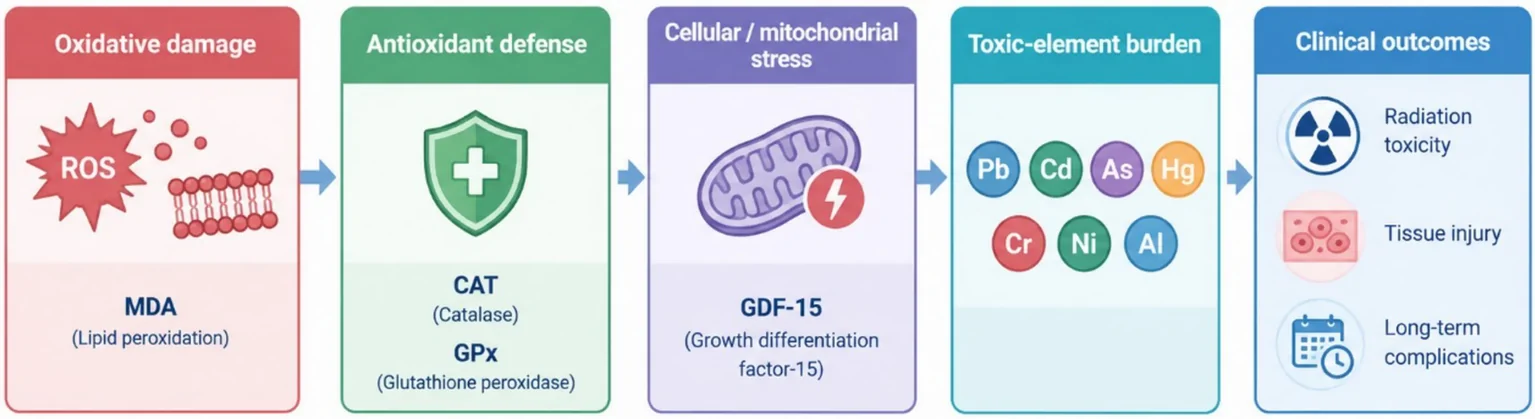
Biomarker framework for evaluating radiation-associated oxidative and cellular stress. Created by the authors using FigureLabs (https://chat.figurelabs.ai/).

Despite its analytical sensitivity, the clinical interpretation of ICP-MS measurements depends on rigorous pre-analytical and analytical quality control. Trace-element measurements in blood, urine, hair, and other biological specimens may be affected by contamination during collection, handling, storage, digestion, and laboratory processing. Contamination from collection devices, reagents, environmental sources, or laboratory materials can produce clinically relevant deviations in measured concentrations, particularly when analytes are present at trace levels. Standardized specimen-collection procedures, contamination-control measures, validated analytical protocols, appropriate reference materials, internal standards, and quality-control procedures are therefore essential. Until such procedures are harmonized and clinically validated reference intervals are established, absolute universal cutoff values for toxic-element burden should be interpreted cautiously.

Despite substantial advances in understanding radiation-induced oxidative stress and mitochondrial dysfunction, an important evidence gap remains concerning the potential contribution of toxic-element exposure to these processes during radiotherapy. Existing studies are characterized by heterogeneity in cancer type, radiation dose and fractionation, treatment combinations, biological specimens, biomarker selection, sampling time points, and analytical methodologies. Furthermore, environmental and occupational exposure, smoking, nutritional status, metabolic disease, and previous anticancer treatment may confound associations between toxic-element burden, oxidative stress, and radiation toxicity. These limitations make it difficult to determine whether toxic elements represent clinically meaningful modifiers of radiation response or primarily reflect broader environmental and biological variability.

Accordingly, this systematic review aimed to synthesize clinical and experimental evidence concerning the relationships among radiotherapy, toxic-element exposure, oxidative stress, mitochondrial dysfunction, and associated biomarkers. Specifically, the review sought to: (1) characterize the biological pathways involved in radiation-induced oxidative and mitochondrial injury; (2) examine evidence linking toxic-element exposure to these pathways; (3) evaluate the reported relevance and limitations of MDA, CAT, GPx, and GDF-15 as biomarkers of radiation-associated biological responses; (4) summarize analytical approaches for toxic-element assessment, with particular emphasis on ICP-MS; and (5) identify methodological limitations and priorities for future translational research. Particular emphasis was placed on evaluating the proposed double-hit framework while distinguishing established biological mechanisms from emerging and clinically unvalidated hypotheses.

## 2 Methods

### 2.1 Study design and framework

This systematic review with narrative synthesis was conducted and reported in accordance with the Preferred Reporting Items for Systematic Reviews and Meta-Analyses (PRISMA 2020) framework. The review was designed to synthesize clinical and experimental evidence concerning radiotherapy-associated oxidative stress, mitochondrial dysfunction, toxic-element exposure, and related biomarkers. Because the included literature encompassed heterogeneous study designs, populations, experimental models, radiotherapy protocols, analytical methods, biomarkers, and outcome measures, quantitative pooling was considered inappropriate. Accordingly, the evidence was synthesized narratively, with findings organized according to biological mechanisms, toxic-element exposure, mitochondrial effects, biomarkers, analytical approaches, and potential clinical implications. The completed PRISMA 2020 checklist is provided as supplementary material. The overall review framework is summarized in the PRISMA 2020 flow diagram (Figure 5).

**Figure 5:**
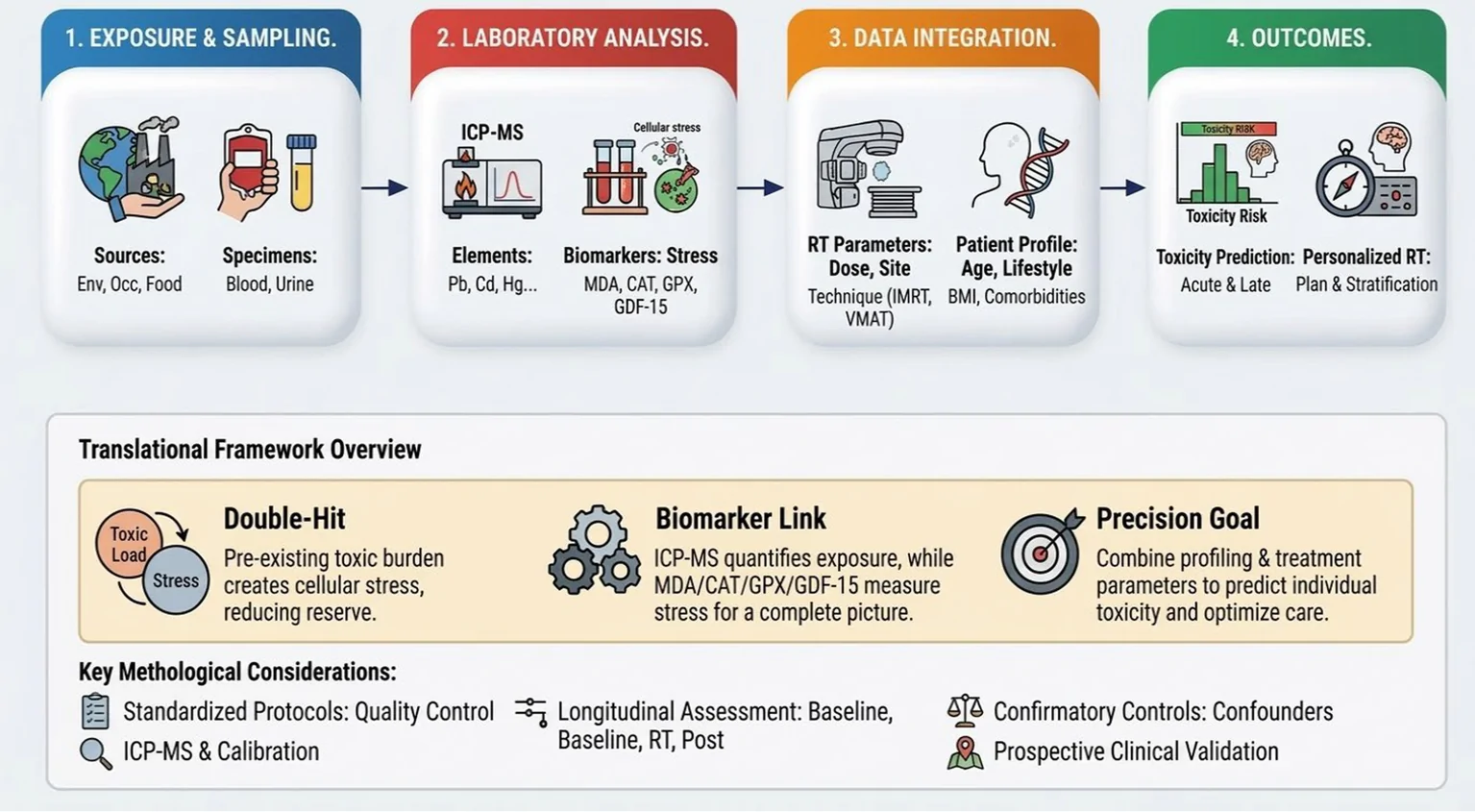
PRISMA flow diagram detailing the systematic literature selection process. Created by the authors using FigureLabs (https://chat.figurelabs.ai/).

### 2.2 Database search strategy and information sources

A comprehensive literature search was conducted in PubMed/MEDLINE, Scopus, Web of Science, and Google Scholar for studies published from January 2000 through December 2025. The search strategy combined controlled vocabulary—including Medical Subject Headings (MeSH), where applicable—with free-text terms addressing three principal concept domains: (1) radiotherapy and ionizing radiation; (2) oxidative stress, mitochondrial dysfunction, and toxic-element exposure; and (3) relevant biomarkers and analytical approaches.

The first domain included terms such as “radiotherapy,” “radiation therapy,” “ionizing radiation,” and “radiation exposure.” The second included “oxidative stress,” “reactive oxygen species,” “mitochondrial dysfunction,” “heavy metals,” “toxic elements,” and the individual elements lead, cadmium, mercury, arsenic, chromium, nickel, and aluminum. The third included “inductively coupled plasma mass spectrometry,” “ICP-MS,” “malondialdehyde,” “MDA,” “catalase,” “glutathione peroxidase,” “CAT,” “GPx,” and “GDF-15.” Boolean operators (AND/OR) were used to combine search concepts. The complete database-specific search strategies are presented in Table 2.

**Table 2:** Detailed database search strategies.

Database	Search strategy
PubMed/MEDLINE	((“Radiotherapy”[Mesh] OR “ionizing radiation” OR “radiation exposure”) AND (“Mitochondrial Dysfunction” OR “Oxidative Stress”[Mesh] OR “reactive oxygen species” OR “heavy metals” OR “toxic elements” OR lead OR cadmium OR mercury OR arsenic OR chromium OR nickel OR aluminum) AND (“Mass Spectrometry”[Mesh] OR ICP-MS OR “malondialdehyde” OR MDA OR catalase OR “glutathione peroxidase” OR “GDF-15”))
Scopus	TITLE-ABS-KEY (“radiotherapy” OR “ionizing radiation” OR “radiation exposure”) AND TITLE-ABS-KEY (“mitochondrial dysfunction” OR “oxidative stress” OR “reactive oxygen species” OR “heavy metals” OR “toxic elements” OR lead OR cadmium OR mercury OR arsenic OR chromium OR nickel OR aluminum) AND TITLE-ABS-KEY (“ICP-MS” OR “malondialdehyde” OR MDA OR catalase OR “glutathione peroxidase” OR “GDF-15”)
Web of Science	TS = (“radiotherapy” OR “ionizing radiation” OR “radiation exposure”) AND TS = (“mitochondrial dysfunction” OR “oxidative stress” OR “reactive oxygen species” OR “heavy metals” OR “toxic elements” OR lead OR cadmium OR mercury OR arsenic OR chromium OR nickel OR aluminum) AND TS = (“ICP-MS” OR “malondialdehyde” OR MDA OR catalase OR “glutathione peroxidase” OR “GDF-15”)
Google Scholar	(“radiotherapy” OR “ionizing radiation”) AND (“oxidative stress” OR “mitochondrial dysfunction”) AND (“toxic elements” OR “heavy metals” OR “ICP-MS” OR “MDA” OR “GDF-15”)

MeSH, Medical Subject Headings; TS, Topic Search; TITLE-ABS-KEY, title, abstract, and keyword fields; ICP-MS, inductively coupled plasma-mass spectrometry; MDA, malondialdehyde; CAT, catalase; GPx, glutathione peroxidase; GDF-15, growth differentiation factor-15.

To identify potentially relevant studies not retrieved through electronic database searching, the reference lists of eligible articles and relevant review publications were manually screened. The database search strategies used for PubMed/MEDLINE, Scopus, Web of Science, and Google Scholar are provided in Table 2.

### 2.3 Study selection and eligibility criteria

Studies were eligible if they evaluated radiotherapy exposure or radiation-induced tissue or cellular injury and reported at least one outcome relevant to the biological framework of this review. Eligible outcomes were classified into two principal domains.

Domain 1: Toxic-element burden and analytical assessment. This included quantitative assessment of toxic or heavy elements, particularly Pb, Cd, Hg, As, Cr, Ni, and Al, using ICP-MS or other validated spectroscopic or analytical methods.

Domain 2: Radiation-associated biological responses. This included mitochondrial bioenergetic parameters, such as ATP production, mitochondrial membrane potential, or respiratory-chain activity; oxidative-stress measures, including ROS, lipid-peroxidation products such as MDA, and antioxidant enzymes such as CAT and GPx; and systemic or cellular stress markers such as GDF-15.

Studies were not required to report outcomes from both domains simultaneously, because the existing literature is fragmented: clinical studies tend to emphasize radiation outcomes and systemic biomarkers, whereas experimental studies more frequently investigate elemental exposure, mitochondrial mechanisms, and subcellular endpoints. The inclusion strategy therefore permitted synthesis of complementary evidence while recognizing that not all included studies directly assessed the combined effects of toxic-element exposure and radiotherapy.

Eligible study designs included randomized controlled trials, non-randomized clinical studies, observational studies, experimental animal investigations, and mechanistic cellular studies. Studies were required to be published in English and to evaluate clinically relevant or experimentally validated models of radiotherapy exposure or radiation-induced injury.

### 2.4 Exclusion criteria

The following publications were excluded: editorials, commentaries, letters, conference abstracts without accessible full text, dissertations, case reports, duplicate publications, and studies unrelated to radiotherapy, ionizing radiation, oxidative stress, mitochondrial dysfunction, toxic-element exposure, or the predefined biomarkers. Studies involving non-ionizing radiation, unrelated toxicities, or unvalidated technical protocols without sufficient biological or clinical relevance were also excluded.

### 2.5 Study selection and screening process

All retrieved records were imported into reference-management software, and duplicate records were removed before screening. Two reviewers independently screened titles and abstracts against the predefined eligibility criteria. Records considered potentially relevant underwent full-text assessment. Disagreements during screening were resolved through discussion and consensus, with consultation of a third reviewer when necessary. The complete study selection process is presented in the PRISMA 2020 flow diagram (Figure 6).

**Figure 6:**
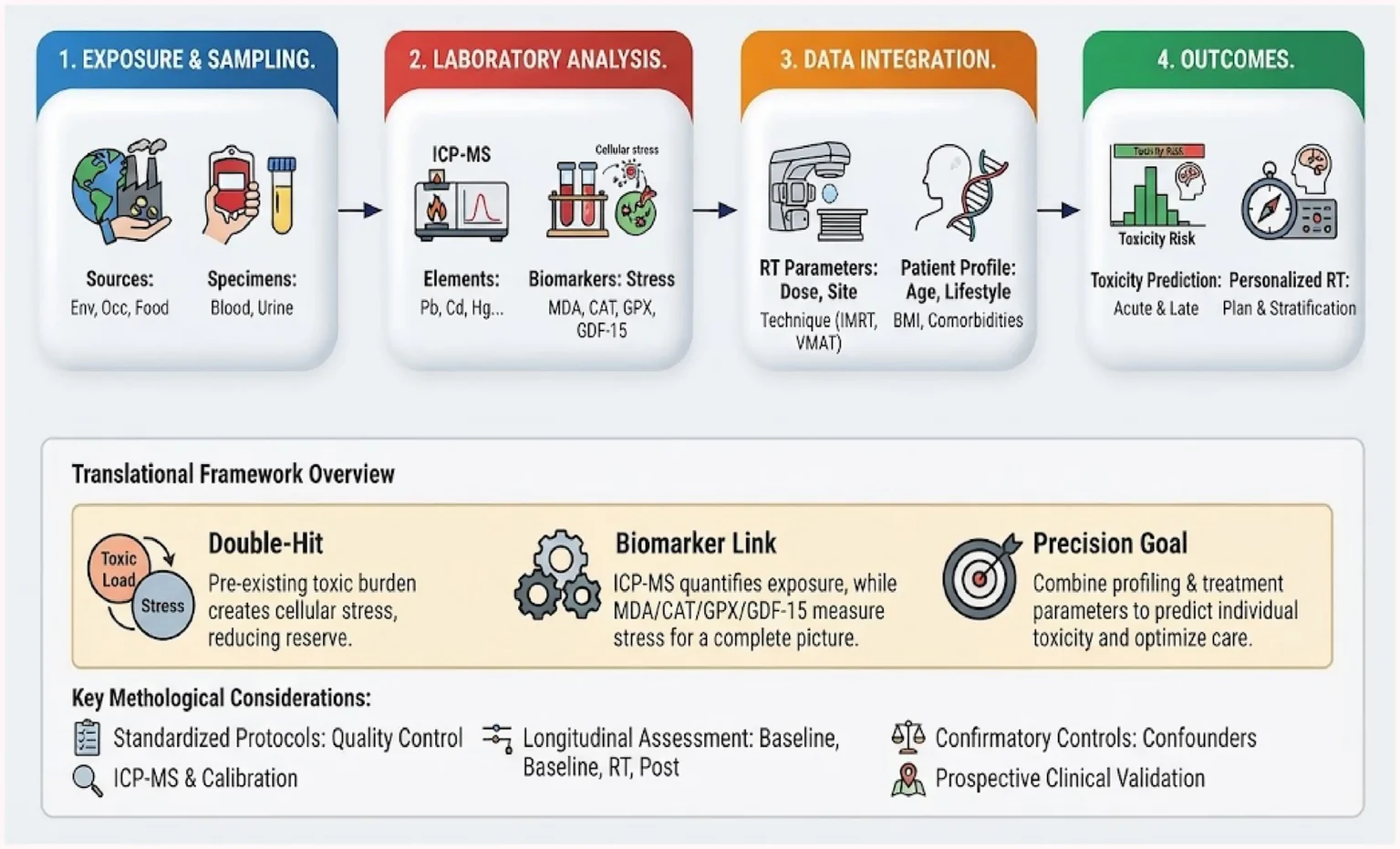
Analytical and translational workflow for toxic-element and biomarker assessment in radiotherapy. Created by the authors using FigureLabs (https://chat.figurelabs.ai/).

### 2.6 Data extraction

Data were extracted independently by two reviewers using a standardized extraction form. The extracted variables included first author, publication year, country, study design, participant characteristics or experimental model, radiotherapy characteristics, toxic elements assessed, analytical methods, biomarkers investigated, biological or clinical outcomes, and principal study findings. Where applicable, information concerning treatment combinations, sampling characteristics, and relevant mechanistic pathways was also recorded.

Discrepancies between reviewers were resolved through discussion and consensus. The extraction framework was designed to facilitate comparison across clinical, animal, and cellular studies while preserving differences in experimental context and outcome definition.

### 2.7 Risk of bias and methodological quality assessment

Methodological quality was assessed according to study design. Randomized controlled trials were evaluated using the Cochrane Risk of Bias 2 (RoB 2) tool. Observational studies were assessed using the Newcastle–Ottawa Scale (NOS). Experimental animal studies were evaluated using the SYRCLE Risk of Bias tool. The methodological-quality assessments were considered when interpreting and weighting the evidence, with greater emphasis placed on findings from studies with fewer methodological concerns. Risk-of-bias assessments were not used as exclusion criteria.

Because cellular mechanistic studies were included in the evidence base, their findings were interpreted primarily as hypothesis-generating evidence rather than as direct evidence of clinical effectiveness or patient-level toxicity.

### 2.8 Narrative evidence synthesis

A qualitative narrative synthesis was performed because substantial heterogeneity prevented meaningful quantitative aggregation. Sources of heterogeneity included differences in cancer type, participant characteristics, experimental models, radiation dose and fractionation, treatment combinations, toxic-element exposure profiles, biological specimens, biomarker definitions, sampling time points, and analytical techniques, including variations in ICP-MS methodology.

The evidence was synthesized according to the following domains:

1. Radiotherapy-induced oxidative stress and redox imbalance;
2. Mitochondrial dysfunction and bioenergetic consequences;
3. Toxic-element-associated oxidative and mitochondrial injury;
4. Potential interaction between toxic-element burden and radiation exposure;
5. Oxidative and cellular stress biomarkers, including MDA, CAT, GPx, and GDF-15;
6. Analytical approaches, particularly ICP-MS-based toxic-element assessment; and
7. Clinical and translational implications, including potential applications in toxicity prediction and personalized radiotherapy.

The synthesis distinguished between evidence derived from human studies and evidence from animal or cellular models. Particular caution was applied when interpreting the proposed interaction between toxic-element exposure and radiotherapy because the available evidence does not establish a clinically validated additive or synergistic effect.

### 2.9 Use of artificial intelligence

Artificial intelligence (AI)-assisted tools were used during manuscript preparation to support structural organization, language refinement, and stylistic editing. AI-generated or AI-assisted text was reviewed and revised by the authors, who verified the scientific content against the underlying literature and retained responsibility for the accuracy, interpretation, and integrity of the final manuscript. The AI tool used during manuscript preparation was Gemini (Google LLC).

## 3 Results

### 3.1 Study selection and evidence base

The systematic search identified 1,420 records across PubMed/MEDLINE, Scopus, Web of Science, and Google Scholar. After removal of 385 duplicates, 1,035 unique records underwent title and abstract screening, of which 867 were excluded. Of the 168 full-text articles assessed, 98 were excluded for predefined reasons: irrelevant clinical outcomes (*n* = 42), absence of relevant toxic-element or ICP-MS data (*n* = 31), and insufficient biomarker information (*n* = 25). Consequently, 74 primary studies fulfilled the eligibility criteria and were included in the qualitative synthesis. Additional review articles, guidelines, and epidemiological reports were consulted for contextual background but were not included in the formal evidence synthesis.

### 3.2 Radiotherapy-induced oxidative stress

Across the included evidence, ionizing radiation was consistently associated with oxidative stress through both direct molecular injury and indirect generation of reactive species. Radiation-induced water radiolysis generates highly reactive species, including hydroxyl radicals, superoxide, and hydrogen peroxide, which can damage DNA, proteins, membrane lipids, and intracellular organelles (5, 9–13, 23, 48).

The reviewed evidence indicated that radiation-induced oxidative stress extends beyond the immediate generation of free radicals. Excessive ROS can oxidize membrane lipids, structural and enzymatic proteins, nucleic acids, and mitochondrial components, thereby disrupting intracellular redox homeostasis and contributing to both tumor-cell killing and normal-tissue injury (12, 13, 23, 28, 37–41, 48).

Hydroxyl radicals were identified as particularly reactive mediators of radiation-associated molecular injury, contributing to DNA strand damage, nucleobase oxidation, DNA–protein crosslinking, and initiation of lipid peroxidation. Hydrogen peroxide, although less reactive, can diffuse across biological membranes and participate in metal-catalyzed reactions that generate highly reactive hydroxyl radicals, thereby propagating oxidative injury (10, 23, 28, 37).

The evidence also demonstrated that oxidative stress has a dual role in radiotherapy. ROS contribute to tumor-cell damage and therapeutic efficacy, while persistent or excessive oxidative imbalance contributes to normal-tissue injury. This duality is consistent with the broader evidence describing ROS as both mediators of cancer-cell death and signaling molecules that may contribute to adaptive stress responses and treatment resistance (13, 49–52).

### 3.3 Mitochondrial dysfunction following radiotherapy

Mitochondrial injury emerged as a recurrent component of the radiation response. The reviewed studies indicated that ionizing radiation can affect mitochondrial membranes, respiratory-chain components, and mitochondrial DNA, with subsequent increases in mitochondrial ROS generation and disruption of oxidative phosphorylation (14–22, 53–56).

Radiation-associated mitochondrial damage was linked to increased electron leakage from the respiratory chain and sustained mitochondrial ROS production. These changes were accompanied by impaired oxidative phosphorylation, ATP depletion, loss of mitochondrial membrane potential, release of pro-apoptotic mediators, and activation of intrinsic apoptotic pathways (14–22, 53–56).

The evidence further connected persistent mitochondrial stress with inflammatory and tissue-remodeling signaling. Crosstalk between ROS and NF-κB signaling, together with mitochondrial injury and altered cellular stress responses, provides a mechanistic basis for persistent inflammation and tissue injury following radiation exposure (20, 21, 55–57). Experimental evidence also indicates that mitochondrial metabolic state can influence tumor radiosensitivity and the cellular response to radiation (15, 16, 22, 58, 59).

Overall, the findings support mitochondrial dysfunction as an important intermediate pathway linking radiation-induced oxidative stress with cellular injury and potentially delayed tissue effects (14–22, 53–59).

### 3.4 Toxic elements and radiation-associated oxidative stress

The included literature identified lead (Pb), cadmium (Cd), mercury (Hg), arsenic (As), chromium (Cr), nickel (Ni), and aluminum (Al) as the principal toxic elements considered in relation to oxidative and mitochondrial injury. Across the evidence base, toxic-element exposure was associated with disruption of antioxidant defenses, altered mitochondrial function, ROS generation, lipid peroxidation, and changes in cellular redox regulation (24–37, 60).

The reported mechanisms differed among individual elements. Lead was associated with impairment of sulfhydryl-containing antioxidant enzymes and disruption of calcium-dependent intracellular signaling (30). Cadmium was associated with glutathione depletion, mitochondrial respiratory-chain impairment, and inflammatory signaling (31). Mercury was linked to thiol-group interactions, enzyme dysfunction, mitochondrial membrane injury, and increased ROS generation (32). Arsenic, chromium, nickel, and aluminum were also associated with oxidative stress, mitochondrial dysfunction, impaired antioxidant defenses, DNA damage, inflammation, and altered cellular energy metabolism (33–37).

These findings demonstrate substantial mechanistic overlap between toxic-element-associated injury and radiation-induced oxidative stress. Both exposure categories can influence antioxidant defenses, ROS generation, lipid peroxidation, mitochondrial function, and DNA integrity (24–29, 37, 60).

However, the reviewed literature largely investigated these pathways independently rather than directly measuring toxic-element burden and radiotherapy response within the same clinical cohorts. Accordingly, the proposed “double-hit” framework remains biologically plausible but incompletely validated. The evidence supports convergence of toxic-element-associated and radiation-associated oxidative pathways, but does not establish that toxic-element exposure consistently produces an additive or synergistic increase in radiotherapy toxicity in cancer patients (24–28, 60).

### 3.5 Oxidative-stress biomarkers

#### 3.5.1 Malondialdehyde

MDA was among the principal biomarkers used to characterize radiation-associated oxidative injury. As a product of polyunsaturated fatty-acid peroxidation, MDA reflects oxidative membrane damage and lipid peroxidation (10–13, 23, 28, 37–41, 48). The reviewed literature associated increased MDA concentrations with enhanced oxidative injury and radiation-associated cellular stress (28, 37, 40, 53–56).

Clinical investigations involving several solid malignancies reported increased serum or plasma MDA following radiotherapy. Persistent elevation was also associated with chronic oxidative stress, inflammatory activation, and delayed tissue injury in some studies (53, 54, 61, 62).

However, MDA is a marker of lipid peroxidation rather than a specific indicator of mitochondrial dysfunction. Its interpretation is therefore strengthened when combined with antioxidant measures such as CAT and GPx (37–41, 61).

#### 3.5.2 Catalase

CAT represents a major enzymatic component of the antioxidant defense system and catalyzes the conversion of hydrogen peroxide into water and molecular oxygen (18, 19, 37–41). The reviewed evidence indicated that radiotherapy may be associated with reduced CAT activity, reflecting increased oxidative burden and impairment or depletion of endogenous antioxidant defenses (37, 40, 53–56, 61).

Reduced CAT activity may permit accumulation of hydrogen peroxide and thereby increase susceptibility to secondary oxidative reactions involving lipids, proteins, DNA, and mitochondrial components (10, 18, 28, 37, 40). However, CAT activity is also influenced by nutritional status, genetic variation, toxic-element exposure, and baseline antioxidant capacity, which may contribute to inter-individual variability (24–27, 30–39).

#### 3.5.3 Glutathione peroxidase

GPx is another major enzymatic component of antioxidant defense. Using reduced glutathione as an electron donor, GPx reduces hydrogen peroxide and lipid hydroperoxides and limits propagation of lipid peroxidation (37–41). Experimental and clinical studies included in the review generally reported reductions in GPx activity following ionizing radiation, consistent with increased oxidative burden and depletion of antioxidant reserves (53–56, 61).

Reduced GPx activity was associated in several studies with increased lipid peroxidation, mitochondrial dysfunction, inflammatory signaling, impaired DNA repair, and delayed cellular recovery (28, 40, 53–56). Because CAT and GPx act through complementary antioxidant pathways, their combined assessment with MDA provides a broader characterization of oxidative damage and antioxidant status than any single biomarker alone (37–41, 61).

### 3.6 GDF-15 and mitochondrial/cellular stress

GDF-15 emerged as a stress-responsive cytokine associated with mitochondrial dysfunction, oxidative stress, inflammation, tissue injury, cellular senescence, and metabolic stress (40, 42–44, 53–58). Studies of mitochondrial disorders have reported increased GDF-15 concentrations in association with mitochondrial dysfunction, supporting its potential value as an indirect marker of mitochondrial stress (42–44).

The available evidence indicates that mitochondrial ROS production, impaired oxidative phosphorylation, mitochondrial DNA damage, and disruption of mitochondrial integrity can activate cellular stress pathways associated with increased GDF-15 expression (14–22, 42–44, 53–58). GDF-15 has also been associated with systemic inflammation, cancer cachexia, metabolic disturbances, and adverse clinical characteristics in several disease contexts (42–45, 63).

However, the evidence specifically evaluating longitudinal GDF-15 changes before and after radiotherapy remains limited. Therefore, GDF-15 should be considered a potential stress-associated biomarker rather than a validated or specific predictor of radiation toxicity. Prospective studies are required to determine its temporal behavior, appropriate reference intervals, and independent prognostic or predictive value during radiotherapy (42–45, 63).

The potential value of combining GDF-15 with MDA, CAT, and GPx lies in their complementary biological domains: GDF-15 reflects broader cellular stress, whereas MDA, CAT, and GPx provide information regarding lipid peroxidation and antioxidant defense (38–44, 53–58, 61, 62).

### 3.7 ICP-MS and toxic-element analysis

ICP-MS emerged as the principal analytical platform for the quantitative determination of trace and toxic elements. Its major analytical advantages include high sensitivity, broad dynamic range, multi-element detection, and high analytical precision (46, 47, 64, 65).

The reviewed evidence indicated that ICP-MS can simultaneously quantify multiple toxic elements, including Pb, Cd, Hg, As, Cr, Ni, and Al, at trace concentrations and is therefore suitable for environmental, occupational, clinical toxicology, and biomedical investigations (46, 47, 64, 65). Compared with conventional atomic absorption spectrometry and ICP-OES, ICP-MS provides lower detection limits and broader multielement analytical capability (46, 47, 64, 65).

Within the context of cancer and radiotherapy research, toxic-element quantification may facilitate investigation of relationships between environmental exposure, oxidative stress, mitochondrial dysfunction, and treatment-associated biological responses (24–27, 46, 47, 60). However, the reviewed evidence does not establish ICP-MS as a biomarker or diagnostic test for radiation toxicity; rather, it is an analytical platform for measuring toxic-element burden.

Analytical reliability depends on appropriate sample collection, preparation, contamination control, calibration, internal standards, reference materials, and quality-control procedures (46, 47, 64, 65). Trace-element contamination during specimen collection, storage, digestion, or laboratory processing may materially influence measured concentrations. Standardization of pre-analytical and analytical procedures is therefore essential for reproducibility and inter-study comparison (46, 47, 64, 65).

### 3.8 Heterogeneity and evidence gaps

Substantial heterogeneity was identified across the included evidence. Differences were observed in cancer type, radiotherapy protocol, radiation dose and fractionation, biological specimen, biomarker selection, analytical methodology, and timing of sample collection (5–8, 42–47, 53–67). These differences limited direct comparison between studies and prevented quantitative pooling.

Patient-related factors also represented important potential sources of heterogeneity. Age, nutritional status, smoking, obesity, diabetes, chronic inflammatory disease, environmental pollution, occupational toxic-element exposure, and previous systemic treatment may independently influence antioxidant capacity and cellular redox homeostasis (24–39, 60). Failure to adequately account for these factors may contribute to variability in reported associations between radiotherapy, oxidative stress, and clinical outcomes (53–56, 60).

The most important evidence gap concerned the direct clinical integration of toxic-element burden with radiotherapy outcomes. Although experimental evidence supports relationships between toxic-element exposure, oxidative stress, and mitochondrial dysfunction, relatively few prospective clinical studies have quantified toxic-element concentrations longitudinally before and after radiotherapy (24–37, 60). Consequently, the clinical significance of toxic-element burden for predicting radiation toxicity, treatment response, and long-term outcomes remains uncertain (53, 60, 68).

A further limitation was the absence of standardized biomarker procedures. Differences in assay methodology, analytical sensitivity, sample preparation, laboratory protocols, and quality-control procedures may contribute to variability in MDA, CAT, GPx, and GDF-15 measurements (42–47, 59–67). The absence of harmonized procedures limits inter-study comparability and currently prevents establishment of universally applicable biomarker thresholds (46, 47, 64, 65, 68, 69).

Finally, the evidence base was characterized predominantly by observational and experimental studies, often involving relatively small populations and limited follow-up. Large, multicenter prospective investigations integrating toxic-element measurements, oxidative-stress biomarkers, mitochondrial indicators, radiation dosimetry, and clinically relevant toxicity outcomes are therefore needed to determine whether these markers have independent predictive or prognostic value (60, 67–69).

## 4 Discussion

### 4.1 Principal findings and interpretation

This systematic review synthesized evidence concerning the relationships among radiotherapy, oxidative stress, mitochondrial dysfunction, toxic-element exposure, and candidate biomarkers of cellular stress. The principal finding was that these processes converge on interconnected biological pathways involving excessive ROS generation, impaired antioxidant defenses, mitochondrial injury, lipid peroxidation, inflammatory signaling, and altered cellular homeostasis (12, 13, 23–28, 37–41, 48, 53–57, 60). However, the evidence base was heterogeneous and included clinical, observational, animal, and cellular studies. Therefore, the findings support a coherent mechanistic framework but do not establish a clinically validated causal relationship between toxic-element burden and radiotherapy toxicity.

While the convergence of toxic-element-induced cellular stress and radiation-generated reactive oxygen species provides strong biological plausibility, direct clinical evidence demonstrating synergistic toxicity in patients remains lacking. Most existing literature evaluates these exposures in isolation rather than tracking baseline metal burdens alongside longitudinal radiation toxicities within unified clinical cohorts. Therefore, describing this framework as a ‘double-hit’ serves to guide future investigative designs and testable hypotheses rather than establishing a clinically proven diagnostic certainty.

The biological plausibility of this framework is strong. Ionizing radiation produces ROS through water radiolysis and radiation-associated cellular reactions, whereas toxic elements can independently impair antioxidant systems, disrupt mitochondrial function, and promote oxidative injury (9–13, 23–37, 48). Mitochondria are positioned at the intersection of these pathways because they are both major sources and important targets of ROS. Radiation-associated mitochondrial injury can increase ROS generation, impair oxidative phosphorylation, reduce ATP production, and contribute to persistent cellular stress (14–22, 53–56). Thus, mitochondrial dysfunction may represent an important mechanistic bridge between acute radiation exposure and prolonged cellular injury.

Importantly, the present review should not be interpreted as demonstrating that toxic elements invariably amplify radiotherapy toxicity. The eligibility criteria permitted inclusion of studies reporting either toxic-element outcomes or radiation-associated biomarkers, and studies were not required to evaluate both exposures simultaneously. Consequently, the evidence supporting interaction between toxic-element burden and radiation remains substantially more limited than the evidence supporting their individual effects.

### 4.2 Oxidative stress as a central mechanism of radiation injury

The reviewed evidence reinforces oxidative stress as a central component of radiation biology. Ionizing radiation produces ROS that can damage DNA, membrane lipids, proteins, and mitochondrial components, thereby contributing to both tumor-cell killing and normal-tissue injury (9–13, 23, 28, 37, 40, 41, 48, 53). This dual effect is clinically important because ROS generation is fundamental to the therapeutic action of radiotherapy, yet persistent oxidative injury may also contribute to treatment-related toxicity.

The findings therefore support a distinction between therapeutically useful oxidative stress and pathological oxidative stress. Tumor cells may be particularly vulnerable to ROS because of altered metabolism, elevated basal oxidative stress, and impaired antioxidant reserve (49–52). Conversely, normal tissues may develop persistent oxidative and inflammatory responses after radiation exposure, contributing to delayed tissue injury and fibrosis (53–57). Therefore, the clinical objective should not be indiscriminate suppression of ROS, because excessive antioxidant intervention could theoretically interfere with tumor-cell killing. Instead, future approaches should focus on identifying patients in whom oxidative injury exceeds the therapeutic window and on selectively modulating pathological pathways.

This distinction also helps explain why antioxidant biomarkers cannot be interpreted simply as measures of treatment efficacy. An increase in oxidative stress may reflect effective tumor-cell injury, normal-tissue toxicity, or both. Biomarker interpretation therefore requires integration with radiation dose, treatment site, tumor response, baseline disease characteristics, and clinically measured toxicity.

### 4.3 Mitochondrial dysfunction as a persistent mechanistic node

A major implication of the reviewed evidence is that mitochondria may function as a mechanistic node linking radiation exposure with persistent oxidative and inflammatory injury. Radiation can induce mitochondrial ROS production, mitochondrial DNA damage, altered membrane potential, impaired oxidative phosphorylation, and disruption of cellular bioenergetics (14–22, 53–56). These effects may sustain oxidative stress after the initial radiation event rather than representing only an acute response.

This finding is particularly relevant because mitochondrial dysfunction may generate a self-reinforcing cycle. Radiation-associated mitochondrial damage can increase electron leakage and ROS production, while ROS can further damage mitochondrial DNA, respiratory-chain components, and mitochondrial membranes (14, 18, 19, 53–56). Persistent mitochondrial dysfunction may subsequently influence inflammatory signaling and cellular fate through pathways involving NF-κB, mitochondrial signaling, and stress responses (20, 21, 57).

The therapeutic implications should nevertheless be interpreted cautiously. Although mitochondrial pathways represent attractive targets, preservation of mitochondrial function in normal tissues must be balanced against the risk of concurrently protecting malignant cells. Evidence supporting mitochondrial-targeted interventions remains primarily mechanistic and translational rather than sufficient to establish routine clinical use (58, 59). Thus, future studies should distinguish interventions that selectively protect normal tissue from those that could compromise tumor radiosensitivity.

### 4.4 cGAS–STING and inflammasome signaling in radiation-induced tissue injury

The cGAS–STING pathway may provide an additional link between radiation-induced DNA damage and persistent inflammation in normal tissues; however, its biological effects are context dependent, because radiation-induced cGAS–STING activation can also contribute to antitumor immune responses. Ionizing radiation can cause nuclear and mitochondrial DNA damage, leading to cytosolic DNA accumulation and activation of cGAS–STING signaling, with subsequent induction of inflammatory responses (60).

Emerging evidence, particularly from studies of radiation-induced lung injury, suggests that cGAS–STING signaling may interact with the NLRP3 inflammasome, promoting inflammatory cytokine production and pyroptotic cell death (60). This pathway may therefore complement the oxidative-stress and mitochondrial framework described in this review, as mitochondrial damage and mtDNA release can further contribute to inflammatory signaling (60).

However, the clinical relevance of cGAS–STING–NLRP3 signaling in radiation toxicity remains insufficiently established. Current evidence is predominantly mechanistic and preclinical, and further prospective studies are required to determine whether these pathways can serve as clinically useful biomarkers or therapeutic targets for radiation-induced normal-tissue injury (60).

### 4.5 Toxic elements as potential modifiers of radiation response

The reviewed evidence supports the concept that toxic elements may contribute to oxidative and mitochondrial stress through mechanisms that overlap with radiation-induced injury. Lead, cadmium, mercury, arsenic, chromium, nickel, and aluminum have been associated with altered antioxidant defenses, ROS generation, mitochondrial dysfunction, lipid peroxidation, and cellular injury (24–37, 67). Individual toxic elements nevertheless differ in their molecular targets and biological behavior, and they should not be treated as a mechanistically uniform group.

Lead and cadmium, for example, have been associated with impairment of antioxidant systems and increased oxidative injury (30, 31). Mercury has a strong affinity for thiol-containing proteins, while arsenic, chromium, nickel, and aluminum have been associated with distinct combinations of oxidative, mitochondrial, inflammatory, and genomic effects (32–37). These differences are important when considering toxic-element burden clinically because total “heavy-metal exposure” may mask substantial variation in the identity, dose, chemical form, duration, and biological distribution of individual elements.

The potential interaction with radiotherapy is biologically plausible because both exposure categories can converge on redox imbalance and mitochondrial injury (24–29, 53, 67). This provides a rationale for the proposed double-hit hypothesis, in which pre-existing toxic-element-associated oxidative stress could reduce cellular reserve before radiation exposure and thereby modify the subsequent biological response.

However, the current evidence does not establish this hypothesis as a clinically demonstrated synergistic phenomenon. Most studies investigating toxic elements and radiation-related oxidative mechanisms did not simultaneously quantify toxic-element burden, radiation exposure, mitochondrial function, and clinical toxicity within the same prospective population. The terms ‘potential interaction’ and ‘double-hit hypothesis’ are therefore more appropriate than describing the relationship as established synergy. This distinction is critical for maintaining appropriate evidentiary rigor.

### 4.6 Clinical interpretation of MDA, CAT, and GPx

The combined biomarker findings suggest that MDA, CAT, and GPx may provide complementary information about radiation-associated redox disturbance. MDA reflects lipid peroxidation, whereas CAT and GPx represent major enzymatic antioxidant defenses (37–41). Their simultaneous assessment may therefore provide a more informative representation of oxidative imbalance than any single marker.

Nevertheless, these biomarkers have limited specificity. MDA can increase in response to numerous pathological and environmental conditions and does not identify the source of oxidative injury. Similarly, CAT and GPx activities are influenced by nutritional status, systemic inflammation, environmental exposures, genetic factors, comorbidities, and baseline antioxidant capacity (24–41). Consequently, changes in these biomarkers should not be interpreted as specific evidence of radiation toxicity without appropriate clinical and biological context.

This limitation has direct implications for biomarker development. Rather than seeking a single universal oxidative-stress marker, future research should evaluate multidimensional biomarker signatures that combine oxidative damage, antioxidant capacity, mitochondrial dysfunction, exposure burden, and clinical outcomes. Such a strategy may provide greater biological specificity and improve discrimination between therapeutic radiation effects and pathological normal-tissue injury.

### 4.7 GDF-15: promising but not yet a radiation-specific biomarker

GDF-15 represents a potentially useful addition to the biomarker framework because it reflects cellular stress and has been associated with mitochondrial dysfunction (42–44). Evidence from mitochondrial disease and systemic inflammatory conditions supports an association between mitochondrial dysfunction and increased GDF-15 concentrations (42–44). GDF-15 may therefore complement MDA, CAT, and GPx by providing information on broader cellular and systemic stress.

However, the present evidence does not justify describing GDF-15 as a radiation-specific biomarker. GDF-15 is influenced by multiple biological processes, including inflammation, cancer-associated metabolic disturbances, tissue injury, and systemic stress (44, 45, 61–64). Elevated concentrations may therefore reflect disease severity or systemic physiological disturbance rather than radiation-induced mitochondrial injury alone.

This distinction is particularly important in oncology because cancer itself may alter GDF-15 concentrations before radiotherapy begins. Future prospective studies should therefore measure GDF-15 longitudinally at standardized time points before, during, and after radiotherapy and should adjust for cancer type, tumor burden, treatment regimen, inflammation, nutritional status, and other major confounders. Only through such designs can the independent predictive value of GDF-15 for radiation toxicity be established.

### 4.8 Temporal dynamics and chronological integration of biomarkers

To effectively translate the selected biomarker panel (MDA, CAT, GPx, and GDF-15) from a conceptual framework into clinically actionable tools, a structured longitudinal sampling strategy is essential. Because biological responses to ionizing radiation evolve across distinct pathological phases, biomarker utility depends heavily on the timing of assessment (65):

- *Pre-treatment baseline*: Evaluating biomarkers prior to the initiation of radiotherapy establishes a critical reference point. This baseline measurement accounts for confounding variables such as pre-existing environmental toxic-element burdens, baseline metabolic stress, systemic inflammation, and lifestyle factors like smoking.
- *During fractionated radiotherapy courses*: Serial sampling during the active treatment course captures acute redox fluctuations. Assessments during this phase reflect immediate water radiolysis, massive reactive oxygen species (ROS) bursts, early lipid peroxidation spikes (measured via MDA), and the acute depletion or compensatory response of enzymatic antioxidant defenses (CAT and GPx).
- *Long-term follow-up*: Evaluations during extended post-treatment recovery windows are vital for monitoring chronic, delayed normal-tissue complications such as tissue fibrosis and organ dysfunction. During this chronic phase, elevated stress-responsive markers like GDF-15 reflect ongoing mitochondrial dysfunction, cellular senescence, and chronic tissue remodeling rather than acute therapeutic toxicity.

Differentiating between these temporal phases ensures that clinicians and researchers do not conflate acute redox shifts with chronic structural injury, thereby improving the diagnostic and prognostic accuracy of biomarker-guided precision oncology.

### 4.9 ICP-MS and the translational toxicological framework

The inclusion of ICP-MS within the proposed framework represents an important methodological strength because toxic-element burden cannot be inferred reliably from oxidative-stress biomarkers alone. ICP-MS provides sensitive multi-element quantification and can simultaneously characterize several potentially relevant toxic elements (46, 47, 68). This makes it particularly suitable for investigations examining environmental or occupational exposure in patients undergoing radiotherapy.

While inductively coupled plasma mass spectrometry (ICP-MS) provides sensitive multi-element quantification, its analytical reliability depends entirely on rigorous pre-analytical controls. Trace-element readings in biological specimens-including blood, urine, and hair- are notoriously susceptible to environmental contamination and procedural inconsistencies during collection, handling, and storage. Minor background trace pollutants from collection tubes, reagents, or laboratory environments can significantly distort heavy-metal values in clinical situations. Consequently, strict standardization of sample collection and contamination-prevention protocols is mandatory. Due to these potential pre-analytical confounders, establishing absolute, universally applicable clinical cutoff thresholds remains premature until rigorous quality assurance guidelines are universally implemented (46, 69).

However, the analytical strength of ICP-MS does not automatically translate into clinical utility. Accurate interpretation requires rigorous control of specimen collection, storage, contamination, digestion, calibration, internal standards, and laboratory quality assurance (46, 47, 65, 69). Trace-element analysis is particularly vulnerable to pre-analytical contamination, which may generate misleadingly elevated concentrations.

A clinically useful framework would therefore require standardized collection and analytical protocols and predefined reference ranges. Importantly, the most informative design would not simply measure toxic elements at one time point. Serial assessment before, during, and after radiotherapy could determine whether baseline exposure predicts subsequent oxidative or mitochondrial responses and whether changes in toxic-element concentrations correspond to treatment-related outcomes.

The combination of ICP-MS with MDA, CAT, GPx, and GDF-15 is consequently best viewed at present as a research framework, rather than a validated clinical testing panel. This distinction should be maintained until prospective studies establish analytical validity, clinical validity, and clinical utility.

### 4.10 Implications for precision radiotherapy

The findings support a broader concept in which radiation response may depend not only on prescribed radiation dose but also on the patient’s pre-existing biological state. Toxic-element burden, antioxidant capacity, mitochondrial function, inflammation, and tumor biology could theoretically contribute to interindividual variation in normal-tissue response (42, 46, 61, 67).

This concept is consistent with the broader movement toward precision radiation oncology, in which biological characteristics are increasingly considered alongside conventional dosimetric parameters (61, 70, 71). A future integrated model could potentially combine radiation dosimetry with toxic-element concentrations, oxidative-stress biomarkers, mitochondrial indicators, clinical characteristics, and imaging or molecular information.

Artificial intelligence and machine-learning approaches may eventually assist with integration of such multidimensional datasets (72). However, predictive modeling should follow, rather than precede, establishment of reliable biomarkers and standardized measurements. Machine-learning models trained on poorly standardized or highly confounded biomarkers may produce apparently accurate predictions without yielding reproducible clinical benefit.

Accordingly, the immediate priority is not the implementation of AI-based toxicity prediction but the development of well-designed prospective cohorts that establish which biological measurements are reproducible, temporally informative, and independently associated with clinically meaningful radiation outcomes.

### 4.11 Modern radiotherapy modalities and radiation-associated biological stress

Advances in radiation delivery, including intensity-modulated radiotherapy (IMRT), volumetric-modulated arc therapy (VMAT), image-guided techniques, stereotactic radiotherapy, and proton therapy, have improved target conformity and can reduce high-dose exposure to selected organs at risk compared with conventional treatment approaches (7, 8, 70, 73, 74). These dosimetric advantages may reduce the volume of normal tissue exposed to clinically relevant radiation doses; however, their effects on systemic oxidative stress, mitochondrial dysfunction, and toxic-element-associated biological responses remain insufficiently characterized (9, 14, 53–57).

IMRT and VMAT may distribute lower radiation doses across larger volumes of normal tissue because of their treatment geometry and beam-delivery characteristics (73). Whether such low-dose exposure produces clinically meaningful persistent oxidative or mitochondrial effects remains uncertain and should not be inferred solely from dosimetric parameters. Similarly, proton therapy can reduce integral dose to normal tissues in selected clinical settings because of its characteristic dose distribution (74), but whether these physical dose advantages consistently translate into reductions in circulating oxidative-stress biomarkers, mitochondrial injury, or long-term normal-tissue toxicity requires prospective comparative investigation.

Future studies should therefore integrate treatment modality, three-dimensional dose distributions, serial oxidative and mitochondrial biomarkers, toxic-element measurements, and clinically defined toxicity outcomes. Such studies would help determine whether reductions in normal-tissue dose achieved with advanced radiotherapy techniques are accompanied by measurable reductions in biological stress.

### 4.12 Strengths and limitations of the evidence base

Several features strengthen the present review. First, the review integrates multiple biological levels, extending from toxic-element exposure and oxidative stress to mitochondrial dysfunction and candidate biomarkers. Second, the inclusion of clinical, animal, and cellular studies permits complementary interpretation of clinical associations and mechanistic pathways. Third, the review incorporates analytical methodology, particularly ICP-MS, which is necessary for translating toxicological concepts into measurable exposure profiles.

Nevertheless, important limitations affect the strength of the conclusions. The principal limitation is heterogeneity. The included studies varied substantially in design, population, cancer type, radiation exposure, experimental model, biological specimen, biomarker selection, analytical procedures, and outcome definitions. This heterogeneity prevented quantitative pooling and limits direct comparison among studies.

A second limitation is that many studies were observational or experimental and involved relatively small samples or limited follow-up. Such designs can demonstrate associations or biological plausibility but cannot reliably establish causality or clinical predictive performance.

Third, toxic-element exposure is strongly influenced by environmental, occupational, nutritional, metabolic, and behavioral factors. Age, smoking, obesity, diabetes, chronic inflammation, pollution, occupational exposure, and previous treatment may independently modify oxidative stress and mitochondrial function (24–39, 60). Inadequate adjustment for these variables may therefore confound associations between toxic-element burden and radiotherapy outcomes.

Fourth, the evidence base does not yet provide standardized thresholds for MDA, CAT, GPx, GDF-15, or toxic-element concentrations that predict radiation toxicity. Finally, the review’s eligibility framework intentionally included studies reporting either toxic-element outcomes or radiation-associated mechanistic biomarkers. Differences in specimen collection, assay procedures, analytical sensitivity, and quality-control practices further limit cross-study comparability (42–47, 59–68).

Finally, the review’s eligibility framework intentionally included studies reporting either toxic-element outcomes or radiation-associated mechanistic biomarkers, but not necessarily both. This broad approach enabled integration of fragmented evidence but also means that the proposed relationship between toxic-element burden and radiotherapy toxicity remains partly inferential. Future studies should directly test this relationship rather than relying primarily on mechanistic convergence.

Compared with previous literature focused predominantly on radiation-induced oxidative stress, mitochondrial dysfunction, or toxic-element toxicity as separate biological phenomena, the present review integrates these domains within a radiotherapy-centered framework. This approach highlights potential biological convergence between radiation-associated redox injury and pre-existing toxic-element-associated cellular stress while maintaining a clear distinction between mechanistic plausibility and clinical validation. However, the available literature remains fragmented, and relatively few studies have simultaneously quantified toxic-element burden, radiation exposure, mitochondrial dysfunction, biomarkers, and clinically defined toxicity outcomes within the same population. Consequently, the proposed framework should be regarded as hypothesis-generating rather than as evidence of a clinically established interaction. Future prospective studies should address this gap through standardized exposure assessment, longitudinal biomarker measurements, radiation dosimetry, and validated clinical toxicity endpoints.

### 4.13 Research priorities and future directions

The next stage of research should move from mechanistic plausibility toward prospective clinical validation. The most important priority is longitudinal assessment of toxic-element burden before, during, and after radiotherapy, combined with serial measurements of oxidative and mitochondrial biomarkers (42–44, 46, 47, 53–61, 66, 67).

A particularly informative study design would simultaneously assess: (i) toxic elements (Pb, Cd, Hg, As, Cr, Ni, and Al); (ii) oxidative stress biomarkers (MDA, CAT, and GPx); (iii) GDF-15; (iv) mitochondrial bioenergetic measures; (v) radiation dose and treatment characteristics; and (vi) clinically defined acute and late toxicities. Such studies would allow investigators to determine whether baseline toxic-element burden predicts subsequent oxidative or mitochondrial responses and whether these biological responses independently predict clinically meaningful radiation injury.

Future investigations should also establish standardized protocols for toxic-element sampling and ICP-MS analysis, including specimen type, collection procedures, contamination prevention, calibration, quality control, reporting standards, and reference intervals (46, 47, 65, 68). Parallel standardization of biomarker assays is necessary before MDA, CAT, GPx, and GDF-15 can be compared reliably across institutions.

Mechanistic studies should further determine whether toxic-element exposure produces an additive, synergistic, or merely overlapping effect with radiation-induced oxidative stress. This distinction cannot be resolved through cross-sectional associations alone and requires experimental designs with controlled exposure conditions and appropriate radiation-only, toxic-element-only, combined-exposure, and control groups.

Finally, translational research should evaluate whether mitochondrial-targeted or redox-modulating interventions can selectively reduce normal-tissue injury without diminishing tumor control (58, 59). Such interventions should be evaluated cautiously because oxidative signaling is also an important component of radiation-mediated tumor-cell killing.

### 4.14 Overall interpretation

Taken together, the evidence supports a biologically coherent relationship among radiation-induced ROS generation, mitochondrial dysfunction, toxic-element-associated oxidative stress, and systemic or cellular stress biomarkers (14–22, 24–44, 53–59, 66, 67). The convergence of these pathways provides a plausible explanation for some of the biological variability observed among patients receiving similar radiation exposures.

However, the evidence currently supports biological plausibility rather than clinical proof of a toxic-element-mediated amplification of radiation toxicity. The “double-hit” hypothesis should therefore be regarded as a testable mechanistic model rather than an established clinical phenomenon.

The most promising translational direction is an integrated approach combining quantitative toxic-element assessment, oxidative-stress biomarkers, mitochondrial indicators, radiation dosimetry, and clinically relevant toxicity endpoints. Such an approach could ultimately contribute to precision radiotherapy, but its clinical implementation requires prospective validation, standardized analytical procedures, rigorous control of confounding, and demonstration of incremental predictive value beyond established clinical and dosimetric factors (61, 67, 69–71).

## 5 Conclusion

Radiotherapy remains one of the most effective therapeutic modalities for cancer management. However, the generation of reactive oxygen species (ROS) is an inevitable consequence of ionizing radiation, leading to oxidative stress and mitochondrial dysfunction in both malignant and normal tissues. Increasing evidence indicates that oxidative stress represents a fundamental biological mechanism underlying radiation-induced cellular injury, inflammation, fibrosis, and treatment-related toxicity. The complex interaction between ionizing radiation and mitochondrial impairment plays a pivotal role in determining cellular radiosensitivity and therapeutic outcomes.

Emerging data suggest that toxic elements may influence the biological response to radiotherapy by disrupting mitochondrial function, impairing endogenous antioxidant defenses, and amplifying oxidative damage. The proposed interaction between radiation-induced ROS production and toxic-element exposure, conceptualized as the “double-hit” hypothesis, provides a valuable mechanistic framework for understanding inter-individual variability in radiation-associated toxicity. However, because this model remains a testable hypothesis rather than a clinically established phenomenon, rigorous longitudinal and prospective human studies are required before it can be applied to clinical risk stratification. Although current evidence remains limited, integration of toxicological assessment into radiotherapy research may offer valuable insights into patient susceptibility and treatment response.

Among the available biomarkers, malondialdehyde (MDA), catalase (CAT), and glutathione peroxidase (GPx) provide valuable information regarding lipid peroxidation and antioxidant status, whereas growth differentiation factor-15 (GDF-15) has emerged as a promising indicator of mitochondrial stress and dysfunction. Simultaneous evaluation of these biomarkers, together with quantitative determination of toxic elements by inductively coupled plasma mass spectrometry (ICP-MS), may facilitate a more comprehensive understanding of radiation-induced biological alterations and support the development of biomarker-guided approaches in clinical oncology.

Despite considerable advances in this field, important knowledge gaps remain regarding the clinical significance of toxic element exposure during radiotherapy, optimal biomarker standardization, and the long-term implications of mitochondrial dysfunction in cancer patients. Well-designed prospective studies with standardized analytical methodologies and larger patient populations are required to validate existing findings and establish clinically applicable biomarker panels.

In conclusion, integrating toxic element analysis, oxidative stress biomarkers, and mitochondrial dysfunction markers represents a promising multidisciplinary approach for advancing precision radiotherapy. Improved understanding of these interconnected biological mechanisms may enhance early detection of radiation-induced toxicity, facilitate individualized treatment strategies, and ultimately improve both therapeutic efficacy and quality of life among patients undergoing radiotherapy.

## Data Availability

The original contributions presented in the study are included in the article/supplementary material, further inquiries can be directed to the corresponding author.

## References

[1] Bray F Laversanne M Sung H Ferlay J Siegel RL Soerjomataram I . Global cancer statistics 2024: GLOBOCAN estimates of incidence and mortality worldwide for 36 cancers in 185 countries. CA Cancer J Clin. (2026):e70090. doi: 10.3322/caac.70090 38572751

[2] Sung H Ferlay J Siegel RL Laversanne M Soerjomataram I Jemal A . Global cancer statistics 2020: GLOBOCAN estimates of incidence and mortality worldwide for 36 cancers in 185 countries. CA Cancer J Clin. (2020) 71:209–49. doi: 10.3322/caac.21660 33538338

[3] Hanahan D. Hallmarks of Cancer: new dimensions. Cancer Discov. (2022) 12:31–46. doi: 10.1158/2159-8290.CD-21-1059, 35022204

[4] Hanahan D Weinberg RA. Hallmarks of Cancer: the next generation. Cell. (2011) 144:646–74. doi: 10.1016/j.cell.2011.02.013, 21376230

[5] Hall EJ Giaccia AJ. Radiobiology for the Radiologist. 9th ed. Philadelphia: Wolters Kluwer (2024).

[6] Delaney G Jacob S Featherstone C Barton M. The role of radiotherapy in cancer treatment: estimating optimal utilization from a review of evidence-based clinical guidelines. Cancer. (2005) 104:1129–37. doi: 10.1002/cncr.21324 16080176

[7] Citrin DE. Recent developments in radiotherapy. N Engl J Med. (2017) 377:1065–75. doi: 10.1056/NEJMra1608986, 28902591

[8] Baskar R Lee KA Yeo R Yeoh KW. Cancer and radiation therapy: current advances and future directions. Int J Med Sci. (2012) 9:193–9. doi: 10.7150/ijms.3635, 22408567 PMC3298009

[9] Azzam EI Jay-Gerin JP Pain D. Ionizing radiation-induced metabolic oxidative stress and prolonged cell injury. Cancer Lett. (2012) 327:48–60. doi: 10.1016/j.canlet.2011.12.012, 22182453 PMC3980444

[10] Ward JF. DNA damage produced by ionizing radiation in mammalian cells: identities, mechanisms of formation, and reparability. Prog Nucleic Acid Res Mol Biol. (1988) 35:95–125. doi: 10.1016/S0079-6603(08)60611-X, 3065826

[11] Spitz DR Azzam EI Li JJ Gius D. Metabolic oxidation/reduction reactions and cellular responses to ionizing radiation. Cancer Metastasis Rev. (2004) 23:311–22. doi: 10.1023/B:CANC.0000031769.14728.bc, 15197331

[12] Schieber M Chandel NS. ROS function in redox signaling and oxidative stress. Curr Biol. (2014) 24:R453–62. doi: 10.1016/j.cub.2014.03.034, 24845678 PMC4055301

[13] Reczek CR Chandel NS. The two faces of reactive oxygen species in cancer. Annu Rev Cancer Biol. (2017) 1:79–98. doi: 10.1146/annurev-cancerbio-041916-065808, 40034143 PMC11875389

[14] Maremonti E Eide DM Rossbach LM Lind OC Salbu B Brede DA. Ionizing radiation induces mitochondrial reactive oxygen species production accompanied by mitochondrial DNA damage and mitochondrial dysfunction. Radiat Res. (2012) 178:425–35. doi: 10.1016/j.freeradbiomed.2019.11.037

[15] Sabharwal SS Schumacker PT. Mitochondrial ROS in cancer: initiators, amplifiers or an Achilles' heel? Nat Rev Cancer. (2014) 14:709–21. doi: 10.1038/nrc3803, 25342630 PMC4657553

[16] Wallace DC. Mitochondria and cancer. Nat Rev Cancer. (2012) 12:685–98. doi: 10.1038/nrc3365, 23001348 PMC4371788

[17] Chandel NS. Navigating Metabolism.Cold Spring Harbor Laboratory Press (2015).

[18] Murphy MP. How mitochondria produce reactive oxygen species. Biochem J. (2009) 417:1–13. doi: 10.1042/BJ20081386, 19061483 PMC2605959

[19] Nolfi-Donegan D Braganza A Shiva S. Mitochondrial electron transport chain: oxidative phosphorylation, oxidant production, and methods of measurement. Redox Biol. (2020) 37:101674. doi: 10.1016/j.redox.2020.101674, 32811789 PMC7767752

[20] West AP Shadel GS. Mitochondrial DNA in innate immune responses and inflammatory pathology. Nat Rev Immunol. (2017) 17:363–75. doi: 10.1038/nri.2017.21, 28393922 PMC7289178

[21] Picard M Shirihai OS. Mitochondrial signal transduction. Cell Metab. (2022) 34:1620–53. doi: 10.1016/j.cmet.2022.10.008, 36323233 PMC9692202

[22] Vyas S Zaganjor E Haigis MC. Mitochondria and cancer. Cell. (2016) 166:555–66. doi: 10.1016/j.cell.2016.07.002, 27471965 PMC5036969

[23] Srinivas US Tan BWQ Vellayappan BA Jeyasekharan AD. ROS and the DNA damage response in cancer. Redox Biol. (2019) 25:101084. doi: 10.1016/j.redox.2018.101084, 30612957 PMC6859528

[24] Valko M Morris H Cronin MTD. Metals, toxicity and oxidative stress. Curr Med Chem. (2005) 12:1161–208. doi: 10.2174/0929867053764635 15892631

[25] Tchounwou PB Yedjou CG Patlolla AK Sutton DJ. Heavy metal toxicity and the environment. EXS. (2012) 101:133–64. doi: 10.1007/978-3-7643-8340-4_6, 22945569 PMC4144270

[26] Jaishankar M Tseten T Anbalagan N Mathew BB Beeregowda KN. Toxicity, mechanism and health effects of some heavy metals. Interdiscip Toxicol. (2014) 7:60–72. doi: 10.2478/intox-2014-0009, 26109881 PMC4427717

[27] Flora SJ Mittal M Mehta A. Heavy metal induced oxidative stress and its possible reversal by chelation therapy. Indian J Med Res. (2008) 128:501–23. 19106443

[28] Valko M Rhodes CJ Moncol J Izakovic M Mazur M Telser J. Free radicals, metals and antioxidants in oxidative stress-induced cancer. Chem Biol Interact. (2006) 160:1–40. doi: 10.1016/j.cbi.2005.12.009, 16430879

[29] Jomova K Valko M. Advances in metal-induced oxidative stress and human disease. Toxicology. (2011) 283:65–87. doi: 10.1016/j.tox.2011.03.001, 21414382

[30] Flora SJS Gupta D Tiwari A. Toxicity of lead: a review with recent updates. Interdiscip Toxicol. (2012) 5:47–58. doi: 10.2478/v10102-012-0009-2, 23118587 PMC3485653

[31] Genchi G Sinicropi MS Carocci A Lauria G Catalano A. The effects of cadmium toxicity. Int J Environ Res Public Health. (2020) 17:3782. doi: 10.3390/ijerph17113782, 32466586 PMC7312803

[32] Bjørklund G Dadar M Mutter J Aaseth J. The toxicology of mercury: current research and emerging trends. Environ Res. (2017) 159:545–54. doi: 10.1016/j.envres.2017.08.051, 28889024

[33] Ratnaike RN. Acute and chronic arsenic toxicity. Postgrad Med J. (2003) 79:391–6. doi: 10.1136/pmj.79.933.391, 12897217 PMC1742758

[34] Costa M Klein CB. Toxicity and carcinogenicity of chromium compounds in humans. Crit Rev Toxicol. (2006) 36:155–63. doi: 10.1080/10408440500534032, 16736941

[35] Das KK Reddy RC Bagoji IB Das S Bagali S Mullur L . Primary concept of nickel toxicity—an overview. J Basic Clin Physiol Pharmacol. (2019) 30:141–52. doi: 10.1515/jbcpp-2017-0171 30179849

[36] Exley C. Aluminum should now be considered a primary etiological factor in Alzheimer's disease. J Alzheimers Dis Rep. (2017) 1:23–5. doi: 10.3233/ADR-170010, 30480226 PMC6159653

[37] Valko M Leibfritz D Moncol J Cronin MTD Mazur M Telser J. Free radicals and antioxidants in normal physiological functions and human disease. Int J Biochem Cell Biol. (2007) 39:44–84. doi: 10.1016/j.biocel.2006.07.001, 16978905

[38] Birben E Sahiner UM Sackesen C Erzurum S Kalayci O. Oxidative stress and antioxidant defense. World Allergy Organ J. (2012) 5:9–19. doi: 10.1097/WOX.0b013e3182439613, 23268465 PMC3488923

[39] Betteridge DJ. What is oxidative stress? Metabolism. (2000) 49:3–8. doi: 10.1016/s0026-0495(00)80077-3 10693912

[40] Sies H. Oxidative stress: a concept in redox biology and medicine. Redox Biol. (2015) 4:180–3. doi: 10.1016/j.redox.2015.01.002, 25588755 PMC4309861

[41] Halliwell B Gutteridge JMC. Free Radicals in Biology and Medicine. 5th ed. Oxford: Oxford University Press (2015).

[42] Montero R Yubero D Villarroya J Henares D Jou C Rodríguez MA . GDF-15 is elevated in patients with mitochondrial diseases and is induced by mitochondrial dysfunction. PLoS One. (2016) 11:e0148709. doi: 10.1371/journal.pone.0148709 PMC4750949 26867126

[43] Ji X Zhao L Ji K Zhao Y Li W Zhang R . Growth differentiation factor 15 is a novel diagnostic biomarker of mitochondrial diseases. Mol Neurobiol. (2017) 54:8110–6. doi: 10.1007/s12035-016-0283-7, 27889897

[44] Tsai VWW Husaini Y Manandhar R Lee-Ng KKM Zhang HP Harriott K . Anorexia/cachexia of chronic diseases: a role for the TGF-β family cytokine MIC-1/GDF15. J Cachexia Sarcopenia Muscle. (2012) 3:239–43. doi: 10.1007/s13539-012-0082-6 PMC3505580 22936174

[45] Valko M Izakovic M Mazur M Rhodes CJ Telser J. Role of oxygen radicals in DNA damage and cancer incidence. Mol Cell Biochem. (2004) 266:37–56. doi: 10.1023/b:mcbi.0000049134.69131.89, 15646026

[46] Thomas R. Practical Guide to ICP-MS: A Tutorial for Beginners. 4th ed. Boca Raton, FL: CRC Press (2013).

[47] Houk RS. Inductively coupled plasma mass spectrometry and its applications. Anal Chem. (1986) 58:97A–105A.

[48] Sies H. Oxidative stress: concept and some practical aspects. Antioxidants (Basel). (2020) 9:852. doi: 10.3390/antiox9090852, 32927924 PMC7555448

[49] Gorrini C Harris IS Mak TW. Modulation of oxidative stress as an anticancer strategy. Nat Rev Drug Discov. (2013) 12:931–47. doi: 10.1038/nrd4002, 24287781

[50] Trachootham D Alexandre J Huang P. Targeting cancer cells by ROS-mediated mechanisms: a radical therapeutic approach? Nat Rev Drug Discov. (2009) 8:579–91. doi: 10.1038/nrd2803, 19478820

[51] Hayes JD Dinkova-Kostova AT Tew KD. Oxidative stress in cancer. Cancer Cell. (2020) 38:167–97. doi: 10.1016/j.ccell.2020.06.001, 32649885 PMC7439808

[52] Klaunig JE. Oxidative stress and cancer. Curr Pharm Des. (2018) 24:4771–8. doi: 10.2174/1381612825666190215121712, 30767733

[53] Liu R Bian Y Liu L Liu L Liu X Ma S. Molecular pathways associated with oxidative stress and their potential applications in radiotherapy. Int J Mol Med. (2022) 49:65. doi: 10.3892/ijmm.2022.5121 PMC8989428 35293589

[54] Szumiel I. Ionizing radiation-induced oxidative stress, epigenetic changes and genomic instability: the pivotal role of mitochondria. Int J Radiat Biol. (2015) 91:1–12. doi: 10.3109/09553002.2014.934929, 24937368

[55] Rong J Yu Q Huang G Wang Y Zhang N. Advances in mitochondrial dysfunction in radiation tissue injury. Front Physiol. (2025) 16:1660330. doi: 10.3389/fphys.2025.1660330, 40964617 PMC12439473

[56] Kim GJ Chandrasekaran K Morgan WF. Mitochondrial dysfunction, persistently elevated levels of reactive oxygen species and radiation-induced genomic instability: a review. Mutagenesis. (2006) 21:361–7. doi: 10.1093/mutage/gel048, 17065161

[57] Morgan MJ Liu ZG. Crosstalk of reactive oxygen species and NF-κB signaling. Cell Res. (2011) 21:103–15. doi: 10.1038/cr.2010.178, 21187859 PMC3193400

[58] Ashton TM McKenna WG Kunz-Schughart LA Higgins GS. Oxidative phosphorylation as an emerging target in cancer therapy. Clin Cancer Res. (2018) 24:2482–90. doi: 10.1158/1078-0432.CCR-17-3070, 29420223

[59] Guo J Xin Q Liu Z Dai S Li J Min Y . Mitochondria-targeted strategies in cancer radiotherapy: from ROS regulation to immunogenic cell death. Front Cell Dev Biol. (2026) 14:1861854. doi: 10.3389/fcell.2026.1861854 PMC13310984 42376458

[60] Li M Ma X Tang Y Huang S Wang H. The role of the cGAS-STING pathway in radiation-induced lung injury: mechanisms and therapeutic perspective. Oncologie. (2026) 28:107–26. doi: 10.1515/oncologie-2025-0373

[61] Wallace DC. A mitochondrial paradigm of metabolic and degenerative diseases, aging, and cancer: a dawn for evolutionary medicine. Annu Rev Genet. (2005) 39:359–407. doi: 10.1146/annurev.genet.39 PMC2821041 16285865

[62] Waris G Ahsan H. Reactive oxygen species: role in the development of cancer and various chronic conditions. J Carcinog. (2006) 5:14. doi: 10.1186/1477-3163-5-14 PMC1479806 16689993

[63] Corre J Hébraud B Bourin P. Concise review: growth differentiation factor 15 in pathology: a clinical role? Stem Cells Transl Med. (2013) 2:946–52. doi: 10.5966/sctm.2013-0055, 24191265 PMC3841089

[64] Emmerson PJ Wang F Du Y Liu Q Pickard RT Gonciarz MD . The metabolic effects of GDF15 are mediated by the receptor GFRAL. Nat Med. (2017) 23:1215–9. doi: 10.1038/nm.4393 28846098

[65] Yang L Ma J Lei P Yi J Ma Y Huang Z . Advances in antioxidant applications for combating 131I side effects in thyroid cancer treatment. Toxics. (2023) 11:529. doi: 10.3390/toxics11060529, 37368629 PMC10303824

[66] Checa J Aran JM. Reactive oxygen species: drivers of physiological and pathological processes. J Inflamm Res. (2020) 13:1057–73. doi: 10.2147/JIR.S275595, 33293849 PMC7719303

[67] Guimarães-Ervilha LO Assis MQ Lopes IDS Iasbik-Lima T Faria JVL Souza ACF . Unravelling Mechanisms of Oxinflammation Induced by Heavy Metals. Metabolites. (2026) 16:619. doi: 10.3390/metabo16050319 PMC13208847 42188028

[68] Becker JS. Inorganic Mass Spectrometry: Principles and Applications. Chichester: John Wiley & Sons (2007).

[69] Rodushkin I Engström E Baxter DC. Sources of contamination and remedial strategies in the multi-elemental trace analysis laboratory. Anal Bioanal Chem. (2010) 396:365–77. doi: 10.1007/s00216-009-3087-z, 19730826

[70] Baumann M Krause M Overgaard J Debus J Bentzen SM Daartz J . Radiation oncology in the era of precision medicine. Nat Rev Cancer. (2016) 16:234–49. doi: 10.1038/nrc.2016.18 27009394

[71] Waterton JC McShane LM O'Connor JPB. Imaging biomarkers exist and they underpin clinical decision-making. Nat Rev Clin Oncol. (2017) 14:452–2. doi: 10.1038/nrclinonc.2017.84 28607515

[72] Bibault JE Giraud P Burgun A. Big data and machine learning in radiation oncology: state of the art and future prospects. Cancer Lett. (2016) 382:110–7. doi: 10.1016/j.canlet.2016.05.033, 27241666

[73] Hall EJ Wuu CS. Radiation-induced second cancers: the impact of 3D-CRT and IMRT. Int J Radiat Oncol Biol Phys. (2003) 56:83–8. doi: 10.1016/S0360-3016(03)00073-7, 12694826

[74] Chang JY Zhang X Wang X Kang Y Riley B Bilton S . Significant reduction of normal tissue dose by proton radiotherapy compared with three-dimensional conformal or intensity-modulated radiation therapy in stage I or stage III non-small-cell lung cancer. Int J Radiat Oncol Biol Phys. (2006) 65:1087–96. doi: 10.1016/j.ijrobp.2006.01.052, 16682145

